# Impacts of delta and omicron variants on inactivated SARS‐CoV‐2 vaccine‐induced T cell responses in patients with autoimmune diseases and healthy controls

**DOI:** 10.1002/ctm2.1171

**Published:** 2023-01-13

**Authors:** Shuyi Wang, Jin Li, Shuang Wang, Yujin Ye, Mengyuan Li, Yihao Liu, Binfeng Chen, Yimei Lai, Liubing Li, Lili Zhuang, Sui Peng, Niansheng Yang, Hui Zhang, Haipeng Xiao

**Affiliations:** ^1^ Department of Rheumatology the First Affiliated Hospital Sun Yat‐sen University Guangzhou China; ^2^ Institue of Precision Medicine the First Affiliated Hospital Sun Yat‐sen University Guangzhou China; ^3^ Department of Endocrinology the First Affiliated Hospital Sun Yat‐sen University Guangzhou China; ^4^ Clinical Trials Unit the First Affiliated Hospital Sun Yat‐sen University Guangzhou China; ^5^ Department of Laboratory Medicine The First Affiliated Hospital of Sun Yat‐sen University Guangzhou China

**Keywords:** cross‐recognition, delta variant, inactivated SARS‐CoV‐2 vaccine, omicron variant, spike‐specific T cell responses

## Abstract

**Background:**

Severe acute respiratory syndrome coronavirus 2 (SARS‐CoV‐2) infection causes coronavirus disease 2019 (COVID‐19), which is still devastating economies and communities globally. The increasing infections of variants of concern (VOCs) in vaccinated population have raised concerns about the effectiveness of current vaccines. Patients with autoimmune diseases (PAD) under immunosuppressant treatments are facing higher risk of infection and potentially lower immune responses to SARS‐CoV‐2 vaccination.

**Methods:**

Blood samples were collected from PAD or healthy controls (HC) who finished two or three doses of inactivated vaccines. Spike peptides derived from wild‐type strain, delta, omicron BA.1 were utilised to evaluate T cell responses and their cross‐recognition of delta and omicron in HC and PAD by flow cytometry and ex vivo IFNγ‐ELISpot.

**Results:**

We found that inactivated vaccine‐induced spike‐specific memory T cells were long‐lasting in both PAD and HC. These spike‐specific T cells were highly conserved and cross‐recognized delta and omicron. Moreover, a third inactivated vaccine expanded spike‐specific T cells that responded to delta and omicron spike peptides substantially in both PAD and HC. Importantly, the polyfunctionality of spike‐specific memory T cells was preserved in terms of cytokine and cytotoxic responses. Although the extent of T cell responses was lower in PAD after two‐dose, T cell responses were boosted to a greater magnitude in PAD by the third dose, bringing comparable spike‐specific T cell immunity after the third dose.

**Conclusion:**

Inactivated vaccine‐induced spike‐specific T cells remain largely intact against delta and omicron variants. This study expands our understanding of inactivated vaccine‐induced T cell responses in PAD and HC, which could have important indications for vaccination strategy.

## Graphical abstract

Inactivated vaccine‐induced‐spike specific T cell cross‐recognize delta and omicron strains.

- Inactivated vaccine‐induced CD4^+^/CD8^+^ T cells respond to delta and omicron variants;
- Polyfunctionality of spike‐specific T cells is preserved in response to delta and omicron variants;
- A third dose of inactivated vaccine expands spike‐specific T cells that recognize delta and omicron variants;
- Spike‐specific T cell responses are lower after two‐dose, but boosted to a greater magnitude by a third dose in PAD.

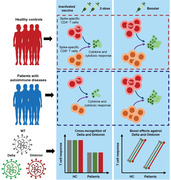

## 1 INTRODUCTION

Severe acute respiratory syndrome coronavirus 2 (SARS‐CoV‐2) spreads throughout the world rapidly, which has caused coronavirus disease 2019 (COVID‐19) pandemic, causing upheavals to socioeconomic well‐being.^1^ SARS‐CoV‐2 vaccines developed under different technologies including mRNA‐, inactivated‐ and adenovirus‐based vaccines, were rapidly developed and widely used across different regions and countries to combat COVID‐19 pandemic. Real‐world data have demonstrated that vaccinations against SARS‐CoV‐2 are of high effectiveness in reducing symptomatic infections and severe outcomes.^2^, ^3^, ^4^ It has also been shown that inactivated vaccines provide protection against COVID‐19 effectively.^5^, ^6^ For their increased transmissibility and immune escape, the emerging variants of concern (VOCs) have posed great challenges in fighting the disease.^7^, ^8^, ^9^, ^10^

Omicron is the most recently identified VOC. By displacing delta variant, omicron variant has been the globally dominant COVID‐19 variant since December 2021.^11^, ^12^ Omicron contains numbers of mutation within spike receptor binding domain (RBD), leading to much higher transmissibility than previous VOCs.^13^ With the mutations in RBD, omicron variant escapes majority of existing neutralising antibodies and are resistance to neutralization.^11^, ^14^ Studies have demonstrated that omicron could escape immune response and undermine the efficacy of current vaccines.^15^, ^16^

The adaptive immune system is important in controlling viral infections. T cells and B cells are the two separate but complementary components that are fundamentally important for vaccination.^17^ Previous studies have observed shared T cell epitopes in SARS‐CoV‐2 variants.^18^ The number of spike‐derived epitopes conserved at 100% amino acid sequence identity constitute majority of T cell epitopes.^19^ Consistently, T cell responses to delta were similar when compared to that of wild type (WT).^20^ However, data about the impacts of VOCs, delta and omicron variants in particular, on inactivated vaccine‐induced T cell responses are limited. There is a large gap in terms of our understanding of T cell responses to inactivated vaccines and their cross‐recognition of delta and omicron variants.

For patients with autoimmune diseases (PAD), the lack of immunomodulation effect of the disease itself and the use of immunosuppressive medications could make them more susceptible to infectious diseases.^21^, ^22^ A previous cohort study reported that patients with chronic inflammatory rheumatic diseases were associated with more severe COVID‐19.^23^ Immunocompromised individuals are more vulnerable to VOCs.^24^ Tacrolimus appeared to diminish T cell responses.^25^ Immune mediating drugs, combination therapy in particular, reduced neutralising antibody responses to inactivated vaccine in patients with rheumatoid arthritis (RA).^26^ Withholding methotrexate after vaccination led to higher anti‐RBD antibody titers compared with continuation of methotrexate in patients with RA or psoriatic arthritis.^27^ Another study showed that interferon gamma (IFNγ)‐specific immune response to the WT or Delta peptides were similar in patients suffered from immune‐mediated inflammatory diseases on immunosuppressive therapy and immunocompetent subjects.^28^ Although humoral responses were lower in patients with inflammatory bowel diseases (IBD) under TNF inhibitors or combination treatments, T cell responses to mRNA vaccines were similar to those of healthy controls (HC).^29^ Nevertheless, there are concerns that vaccine efficacies could be potentially attenuated in PAD.

Our previous study reported that the third dose of inactivated vaccine enhanced T cell responses against WT strain significantly.^30^ Whether a boost vaccine could enhance T cell responses and their cross‐recognition of delta and omicron in PAD remain to be explored. In this study, we focused on spike‐specific T cell responses to WT, as well as delta and omicron variants after two‐dose and three‐dose of inactivated vaccine in HC and PAD.

## 2 METHODS AND MATERIALS

### 2.1 Study design and participants

In cohort 1, 56 PAD (systematic lupus erythematosus (SLE, *n* = 29), RA (*n* = 11), primary Sjogren's syndrome (pSS, *n* = 7), ankylosing spondylitis (AS, *n* = 9)) were recruited between January 2022 and October 2022, from the First Affiliated Hospital of Sun Yat‐sen University (FAH‐SYSU) in Guangzhou, China. Gender‐ and age‐matched HC (*n* = 25) were recruited accordingly. All participants in this cohort have finished two‐dose of inactivated vaccine. Blood samples were collected at a mean of 116 days in PAD and 120 days in HC after the second dose. To further assess T cell responses and their cross‐recognition of delta and omicron induced by prime vaccination and a boost dose of inactivated vaccine in PAD as well as HC, a second cohort was recruited. In cohort 2, 28 PAD (SLE, RA, pSS and AS) and 24 gender‐ and age‐matched HC were recruited between January 2022 and October 2022, from FAH‐SYSU. Each participant donated blood sample right before the third dose of vaccine (PAD received boost vaccine at a mean of 206 days and HC at a mean of 205 days after the second vaccination) and 4 weeks after the boost, respectively. We were able to collect blood samples from 18 PAD and 11 HC after the third dose. Tables S1 and S2 displayed the demographics and clinical characteristics of the participants. Informed written consent was signed by all participants. Ethical approval was given by the FAH‐SYSU (2021 850).

### 2.2 Cell isolation

Venous blood was collected in heparinised tubes. Blood was diluted with two times volume of PBS and slowly loaded on top of Ficoll (Stemcell, Cat#07861). Samples were centrifuged at 1500 rpm for 22 min and layer with peripheral blood mononuclear cells (PBMCs) was carefully collected. Cells were then washed twice with PBS and cryopreserved in freezer media. Samples were placed in a freezing container at −80°C overnight and then transported to liquid nitrogen.

### 2.3 Peptides

Peptide pools derived from WT strain, Delta and Omicron BA.1 variants were used to detect spike‐specific T cells. The peptide pools are divided into two subpools of 158&158 (or 157) peptides, including 316 or 315 peptides made from the whole spike glycoprotein of SARS‐CoV‐2 by peptide scanning (15mers with 11 aa overlap) (GenScript).

### 2.4 **Activation** induced mar**ker expression assays**

Activation induced marker (AIM) expression assays were conducted using PBMCs as described.^31^ PBMCs were thawed and diluted with complete media (containing 10% fetal bovine serum). Cells were washed and suspended at a density of 2.5 × 10^6^/ml. For each condition, 200 μl complete media containing 0.5 × 10^6^ PBMCs were let to rest in 96‐well plates overnight. After resting, cells were stimulated with spike peptide pools (1 μg/ml, MabTech, Cat#3630‐1) and anti‐human CD28 antibody (2 μg/ml, BioLegend, Cat#302934). Matched control samples for each donor were treated with an equal amount of DMSO. Cells were washed after 24 h stimulation. Cells were surface stained with fluorophore conjugated FACS antibodies against CD3, CD4, CD8, CD45RA, CCR7, CD69, OX40, 4‐1BB, CXCR3, CXCR5, CD95, and CD107a for 20 min. Zombie Red was used for dead cell exclusion.

### 2.5 Intracellular cytokine staining

Intracellular cytokine staining (ICS) was conducted as described previously.^32^ Spike peptide pools derived from SARS‐CoV‐2 WT (Genscript, Cat#RP30020), Delta (Genscript, Cat#RP30033), and Omicron BA.1 (Genscript, Cat#RP30121) were utilised to assess spike‐specific T cell responses. Thawed PBMCs were let to rest overnight. Cells were stimulated with spike peptides (1 μg/ml) in the presence of anti‐human CD28 antibody (2 μg/ml) for 20 h. Golgi‐Plug containing brefeldin A (Sigma–Aldrich, Cat#B7651) was added to stop cytokine from secretion for additional 4 h. After surface staining performed as described above, fixation and permeabilization were performed using fixation/permeabilization buffer (BD Bioscience, Cat#554714) for 30 min. Cells were stained with antibodies against IFNγ, interleukin‐2 (IL‐2) and tumor necrosis factor α (TNFα) at 4°C for 30 min, followed by washing and suspension prior to data acquisition.

### 2.6 Flow cytometry

All flow cytometry samples were loaded and acquired on 5 laser Cytek™ AURORA. We performed FACS data analysis by FlowJo (Tree Star, USA) software. Details of all antibodies and peptides were available in Tables S3 and S4, respectively. Figures S1 and S2 show the gating strategies for T cell subsets and cytokine expression, respectively.

All data were subtracted from background using paired DMSO samples. After background‐subtracted, only samples with level of AIM^+^ CD4^+^ or CD8^+^ T cells > 0.05%, as well as frequency of cells producing cytokine > 0.01% was defined as positive response to spike peptide pool stimulation as described.^31^, ^33^, ^34^ For cell subsets analysis, the AIM^+^ background was subtracted independently. The definitions of AIM^+^ CD4^+^ and CD8^+^ T cells were referred to dual‐expression of OX40 and 4‐1BB or CD69 and 4‐1BB.^31^

### 2.7 Ex vivo ELISpot

To detect IFNγ‐secreting T cells, ex vivo ELISpot assay was performed.^30^ All the reagents used in ELISpot assay were listed in Table S5. For precoating, plates were incubated with anti‐human IFNγ antibody (MabTech, Cat#3420‐3‐250) in 100 μl PBS at a concentration of 15 μg/ml at 4°C overnight. The precoated plates were then washed 4–5 times to remove the unbound antibodies completely. PBMCs (2×10^5^/well) were seeded to the wells with spike peptides (1 μg/ml) for 28 h. Cells stimulated with phytohemagglutinin (Sigma–Aldrich, Cat#P8139) and Ionomycin (Sigma–Aldrich, Cat#I0634) were used as positive controls. After stimulation, the wells were washed 4–5 times and incubated with biotinylated anti‐IFNγ antibody for 2 h. Wells were then incubated with Horseradish Peroxidase Avidin D for 1 h to detect positive spots of IFNγ‐secreting T cells. Responses against spike peptide pool were considered positive if the ELISpot count results ≥25 spot‐forming units (s.f.u.)/10^6^ PBMCs.

### 2.8 Statistical analysis

Prism 8.0 was used to perform statistical analysis. In the comparison between HC and PAD, Mann–Whitney *U*‐test or Student's *t*‐test were implemented as appropriate. Wilcoxon‐signed rank *t*‐test was used to evaluate significance decreases of fold‐change for VOCs with a hypothetical median of 1. As for the comparison of fold‐change between pre‐boost and post‐boost, Wilcoxon matched‐pairs signed rank test was used. Correlations were performed via linear regression. Two‐tailed *p* < 0.05 was considered statistically significant in this study.

## 3 RESULTS

### 3.1 Study participants

In both cohorts, no participants had a clinical history of, or the presence of SARS‐CoV‐2 infection. Disease activity scores were not affected by inactivated SARS‐CoV‐2 vaccination. Severe adverse events to vaccination were not observed in any of the participants (Tables S1 and S2). CD4^+^ and CD8^+^ T cells accounted for 38.15% and 26.15% in PAD, 40.90% and 24.40% in HC in the PBMCs, respectively. Phenotypically, we observed lower frequencies of CD45RA^−^CCR7^+^ CD4^+^ and CD8^+^ central memory T cells (T_CM_) in PAD. CD4^+^ and CD8^+^ T_CM_ were 28.8% and 3.3% in PAD, 39.0% and 4.4% in HC. The frequency of CD45RA^−^CCR7^−^ CD4^+^ effector memory T cells (T_EM_) was 1.2 times higher in PAD than HC. The percentage of CD45RA^+^CCR7^−^ CD8^+^ terminally differentiated T cells (T_EMRA_) was 1.3 times higher in PAD than HC (Figure S3).

### 3.2 Patients with autoimmune diseases **show lower CD4+ T cell responses after** two**‐dose of inactivated vaccine**

**FIGURE 1:**
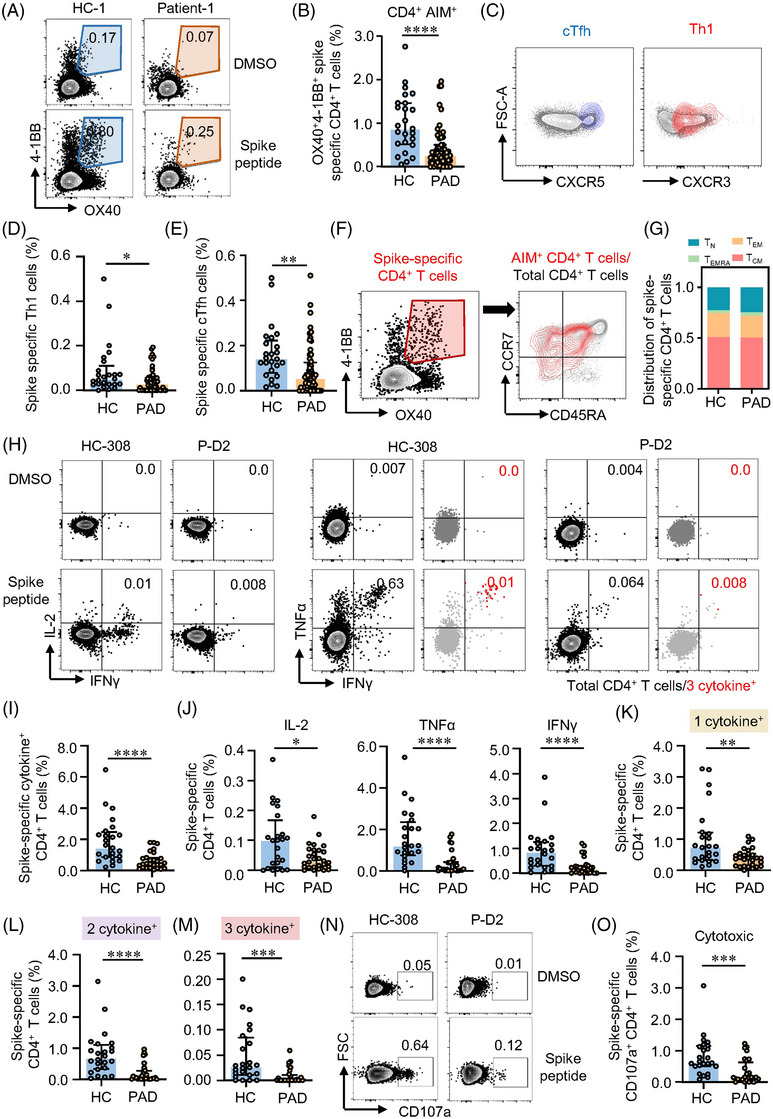
Two‐dose of inactivated vaccine induces lower spike‐specific CD4^+^ T cells responses in patients with autoimmune diseases (PAD). Peripheral blood mononuclear cells (PBMCs) from PAD or healthy controls (HC) who received two‐dose of inactivated vaccine were stimulated by wild‐type (WT) spike peptide pools. Spike‐specific CD4^+^ T cells were assessed by the expression of activation‐induced markers (AIM) and cytokine production in response to spike peptide pool stimulation by flow cytometry. (A, B) Percentages of spike‐specific AIM^+^ (OX40^+^4‐1BB^+^) CD4^+^ T cells in PAD or HC. Representative plots are shown. (C) Representative plots showing the expression of CXCR3 and CXCR5. Blue events depict CD4^+^ AIM^+^ cTfh cells (CXCR5^+^) and red events depict CD4^+^ AIM^+^ Th1 cells (CXCR3^+^CXCR5^−^). Grey events represent total CD4^+^ T cells from the same donors. (D, E) Comparisons of CD4^+^ AIM^+^ Th1 and cTfh cells between HC and PAD, respectively. (F) Flow cytometric plots depicting memory subsets of spike‐specific CD4^+^ T cells. Red events depicting AIM^+^ CD4^+^ T cells. (G) Distribution of memory AIM^+^ CD4^+^ subsets in HC and PAD. (H) Representative flow cytometric plots showing cytokine production in CD4^+^ T cells stimulated by spike peptide pools. Spike‐specific CD4^+^ T cells were divided into 1 cytokine^+^ (producing anyone of IFNγ, TNFα and IL‐2), 2 cytokines^+^ (producing any two of IFNγ, TNFα and IL‐2) and 3 cytokines^+^ (producing all 3 cytokines) spike‐specific CD4^+^ T cells. Red events depicting CD4^+^ T cells producing all 3 cytokines. (I) Comparison of CD4^+^ T cells producing at least one cytokine between HC and PAD. (J) Percentages of IL‐2‐, TNFα‐ and IFNγ‐producing spike‐specific CD4^+^ T cells in HC and PAD. (K‐ M) Comparisons of CD4^+^ T cells producing 1 cytokine, 2 cytokines and 3 cytokines between HC and PAD. (N, O) Representative plots depicting CD107a expression in CD4^+^ T cells stimulated by spike peptide pools. Frequencies of CD107a^+^ CD4^+^ T cells in HC and PAD were summarised. Data are expressed as median with interquartile range. HC = 25, PAD = 56 in panels B, D, E, G. HC = 24, PAD = 28 in panels I, J, K, L, M, O. *p < .05, **p < .01, ***p < .001, ****p < .0001 by Mann–Whitney test. T_N_: naïve T cells. T_EM,_ effector memory T cells. T_CM_, central memory T cells. T_EMRA_, terminally differentiated T cells.

SARS‐CoV‐2 vaccination related serological response is impaired in patients with PAD as shown in previous studies.^35^, ^36^ We were to assess T cell responses to inactivated vaccines in PAD. Cells were stimulated with WT spike peptide pools to test the level of spike‐specific T cells and their functional status. In consistent with our previous data,^30^ AIM^+^ CD4^+^ T cells were detectable in all HC samples nearly 4 months after two‐dose of inactivated vaccine. While 9 out of 56 samples from PAD showed no responses to spike peptide stimulation (Table S6). AIM^+^ CD4^+^ T cells specific to spike peptides were significantly lower in PAD (Figure 1A,B). Although the level of AIM^+^ CD4^+^ T cells was similar across different diagnosis, either mono‐ or combination‐therapy posted great impact on T cell responses to vaccination (Figure S4). Given the important roles of T follicular helper (Tfh) cells in vaccination and T helper 1 (Th1) cells in viral infection, we examined spike‐specific circulating Tfh (cTfh, CXCR5^+^) and Th1 (CXCR5^−^CXCR3^+^) cells.^32^ Consistent with previous study in mRNA vaccine,^32^ inactivated vaccine‐induced AIM^+^ CD4^+^ T cells were dominated by CXCR5^+^ cTfh cells and CXCR5^−^CXCR3^+^ Th1 cells in both PAD and HC (Figure 1C). Notably, the frequencies of spike‐specific cTfh and Th1 cells were lower in PAD than HC (Figure 1D,E), while the proportions of these helper cells within AIM^+^ T cells were similar (Figure S5A,B), suggesting that T helper cell differentiation was not affected. Phenotypically, AIM^+^ CD4^+^ T cells were mostly T_CM_ and T_EM_ cells in both PAD and HC (Figure 1F), which resembled the phenotype of nature SARS‐CoV‐2 infection.^31^ Although the absolute percentages of memory T cell subsets were lower in PAD, the proportions of these memory subsets were similar between PAD and HC (Figure 1G, Figure S5C). Similar results were found in Cohort 2 (Figure S6A–D). Interestingly, we detected a cluster of AIM^+^ CD4^+^ T cells with the phenotype of stem cell‐like memory T cells (T_SCM_) (CD45RA^+^CCR7^+^CD95^+^) in both PAD and HC. Not surprisingly, AIM^+^ CD4^+^ T_SCM_ were also lower in PAD than HC (Figure 1F, Figure S6E). As a cluster of minimally differentiated memory T cells, T_SCM_ could maintain life‐long immunological memory.^37^ These data indicate that two‐dose of inactivated vaccine induced a lower magnitude of CD4^+^ T cells memory against SARS‐CoV‐2 in PAD.

ICS was performed to further explore CD4^+^ T cell responses in PAD and HC. The polyfunctionality of spike‐specific T cells was measured by combination of IL‐2, TNFα and IFNγ, as well as surface marker CD107a for cytotoxic T cells (CTL). In ICS analysis, spike‐specific CD4^+^ T cells were categorised into 1 cytokine^+^, 2 cytokine^+^ and 3 cytokine^+^ based on the kinds of cytokine produced (Figure S2). We found that 2 cytokine^+^ and 3 cytokine^+^ were detected in these CD4^+^ T cells in both groups under spike peptide stimulation (Figure 1H–1M), indicating the polyfunctional capability of these spike‐specific CD4^+^ T cells from two‐dose inactivated vaccination. Consistent with AIM expression assay, CD4^+^ T cells producing at least one cytokine (cytokine^+^) was lower in PAD compared with HC (Figure 1I). Looking into each cytokine, the percentages of IL‐2, TNFα and IFNγ were all lower in PAD compared to HC (Figure 1J). Spike‐specific CD4^+^ T cells in PAD not only showed less cytokine production, their polyfunctionality was also impaired (Figure 1K–1M). Besides, spike‐specific CD4^+^ CTL marked by CD107a,^38^ was also lower in PAD than in HC (Figure 1N,O), suggesting less cytotoxic effects of these specific CD4^+^ T cells. Together, two‐dose of inactivated vaccine induced long‐lasting memory CD4^+^ T cells associated with multifunction in both PAD and HC. However, spike‐specific CD4^+^ T cells were detected lower in PAD by two‐dose of inactivated vaccine.

### 3.3 Patients with autoimmune diseases **show lower CD8^+^ T cell responses after vaccination**

**FIGURE 2:**
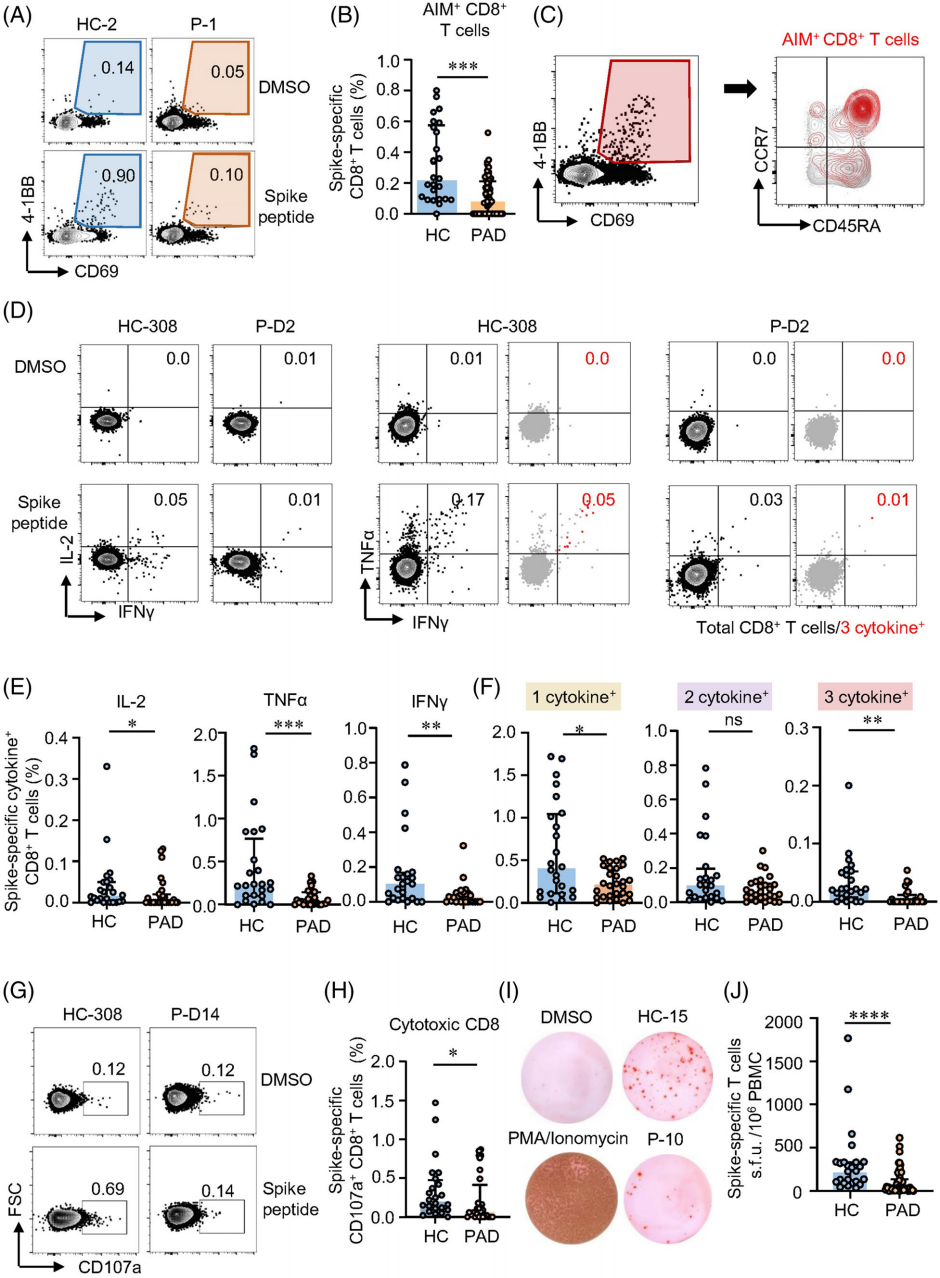
Two‐dose of inactivated vaccine elicits lower spike‐specific CD8^+^ T cell responses in patients with autoimmune diseases (PAD). Peripheral blood mononuclear cells (PBMCs) from PAD or healthy controls (HC) who received 2‐dose of inactivated vaccine were stimulated with spike peptide pools as in Figure 1. Spike‐specific memory CD8^+^ T cells and cytokine response to SARS‐CoV‐2 were evaluated by Activation induced marker (AIM) and Intracellular cytokine staining (ICS) through flow cytometry. (A) Representative plots showing spike‐specific CD8^+^ AIM^+^ T cells in PAD or HC. (B) Comparison of spike‐specific AIM^+^ CD8^+^ T cells between in PAD or HC. (C) Flow cytometric plots showing memory subsets of CD8^+^ T cells. Red events depict spike‐specific AIM^+^ CD8^+^ T cells. (D) Flow cytometric plots showing cytokine production in CD8^+^ T cells stimulated by spike peptides. Red events depict CD8^+^ T cells producing all 3 cytokines. (E) Percentages of IL‐2‐, TNFα‐ and IFNγ‐producing CD8^+^ T cells in HC and PAD were summarised. (F) Comparisons of CD8^+^ T cells producing 1 cytokine, 2 cytokine and 3 cytokines between PAD or HC. (G, H) Cells were stimulated with spike peptide pools and SARS‐CoV‐2‐specific cytotoxic CD8^+^ T cells were measured by flow cytometry for the expression of CD107a. Representative plots were shown and data were summarised in (H). (I, J) SARS‐CoV‐2‐responsive T cells were measured by IFNγ ELISpot. Representative images of ELISpot wells were shown. The numbers of IFNγ‐producing T cells were quantified by calculating the spot‐forming units (s.f.u.) and summarised in (J). HC = 25, PAD = 56 in panels B, J. HC = 24, PAD = 28 in panels E, F, H. Data are expressed as median with interquartile range. *p < .05, **p < .01 and ***p < .001 by Mann–Whitney test. ns: not significant.

CD8^+^ T cells are critical in viral clearance.^39^ To evaluate CD8^+^ T cell responsiveness to inactivated vaccine, AIM assay and ICS were performed on samples from PAD and HC. All HC but only 60.7% (34/56) of PAD generated spike‐specific AIM^+^ CD8^+^ T cells (Table S7). SLE, RA, AS and pSS showed similar percentages of AIM^+^ CD8^+^ T cells, but significant lower compared to HC (Figure S4, Figure 2A,B). Differently, AIM^+^ CD8^+^ T cells were mainly composed by T_EMRA_ and CD45RA^+^CCR7^+^ subsets (Figure 2C, Figures S5D, S6F–S6H). Majority of CD45RA^+^CCR7^+^ CD8^+^ T cells expressed CD95 and could be further defined as T_SCM_ (Figure S6F). Consistently, all the memory CD8^+^ T cell subsets were detected lower in both cohorts of PAD compared to HC (Figures S5D and S6G).

The expression of IL‐2, TNFα and IFNγ in response to spike peptides was used to assess the functionality of spike‐specific CD8^+^ T cells. Inactivated vaccine‐elicited CD8^+^ T cells were also “equipped” with polyfunctional potency for the detectable level of CD8^+^ T cells producing more than 1 cytokine in both PAD and HC (Figure 2D). The percentages of IL‐2, TNFα and IFNγ were all lower in PAD compared to HC (Figure 2E). Notably, these AIM^+^ CD8^+^ T cells producing 1 cytokine or 3 cytokines were also lower in PAD (Figure 2F). Moreover, PAD showed lower level of spike‐specific CD107a^+^ cytotoxic CD8^+^ T cells (Figure 2G,H). Next, we applied ex vivo ELISpot to further assess cellular functions of spike‐specific T cells. In the ex vivo IFNγ ELISpot experiments, all the samples from HC were detected positive whereas only 67.9% samples from PAD were detected positive (Table S8). Among the responders, we also found significantly fewer spike‐specific T cells producing IFNγ in PAD (Figure 2I,J). Taken together, these data demonstrate that two‐dose inactivated vaccine induce robust CD8^+^ T cells with polyfunctionality but lower in PAD.

### 3.4 **Inactivated vaccine‐induced CD4^+^ and CD8^+^ T cells are highly conserved to** d**elta and** o**micron variants**

Many studies have demonstrated that neutralising activities of sera from vaccinees against VOCs are substantially reduced.^40^ The currently dominant SARS‐CoV‐2 strain omicron are resistant to neutralising antibody responses.^41^ We were to explore T cell cross‐recognition of the recent VOCs including delta and omicron in PAD and HC. We used AIM assay and ICS to characterise spike‐specific memory T cell responses and calculated each variant recognition as fold‐change relative to WT (only samples owning positive response to WT strain were analysed).

**FIGURE 3:**
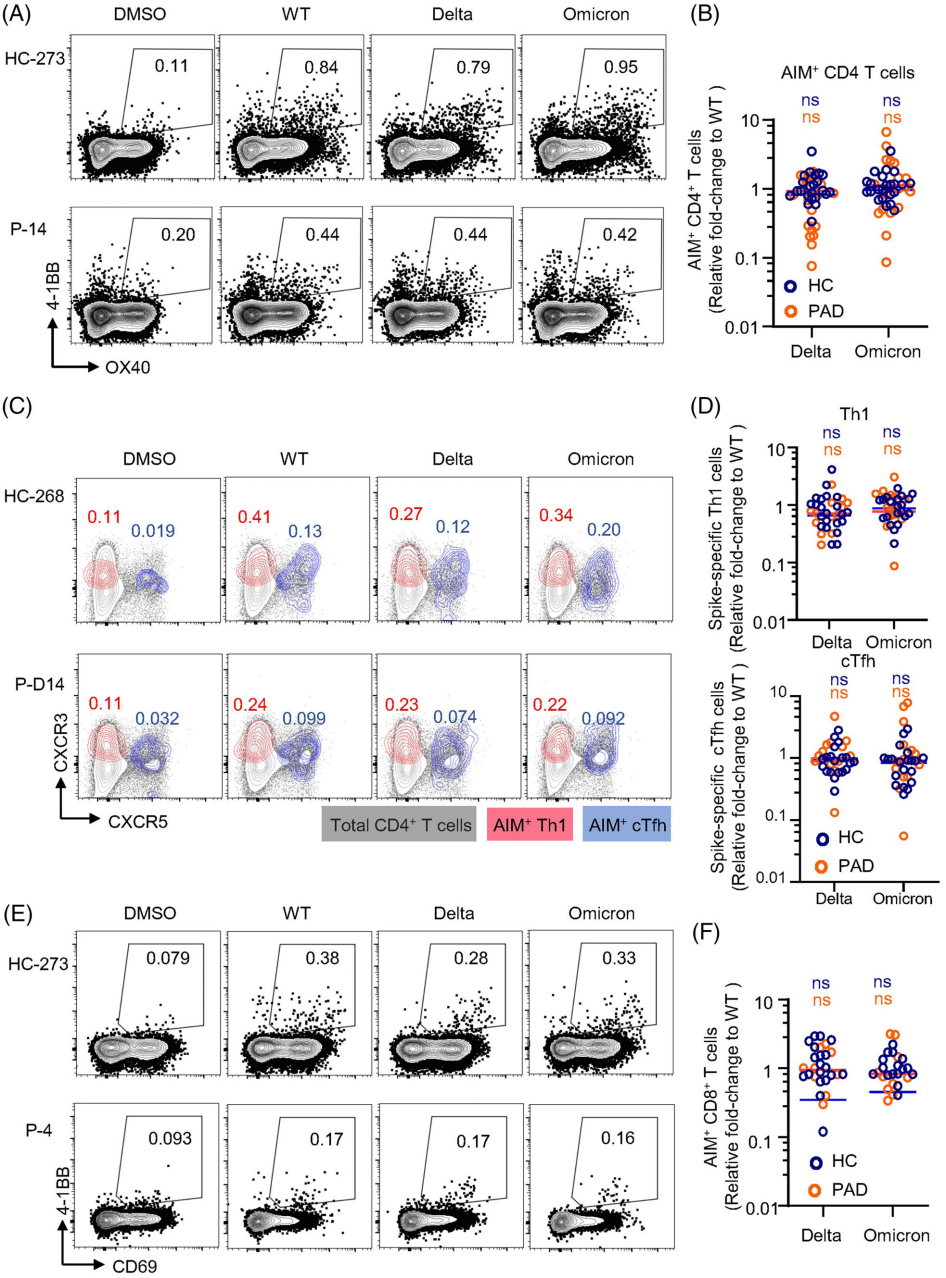
Impacts of variant‐associated mutations on inactivated vaccine induced spike‐specific memory T cell recognition. Spike‐specific memory T cells from fully vaccinated patients with autoimmune diseases (PAD) or healthy controls (HC) were assessed for their recognition of delta and omicron variants. Cells were stimulated with spike peptide pools of wild‐type (WT), delta and omicron BA.1. T cell reactivity to variants was relative to WT. (A) Representative flow cytometric plots depicting the gating of AIM^+^ (OX40^+^4‐1BB^+^) CD4^+^ T cell responses to WT, delta or omicron BA.1 spike peptide pools. (B) Relative fold‐change of CD4^+^ AIM^+^ T cell cross‐recognition of delta and omicron to WT (HC = 23, PAD = 20). (C) Representative flow cytometric plots showing AIM^+^ Th1 (CXCR3^+^CXCR5^−^) and AIM^+^ cTfh (CXCR5^+^) cells that recognized spike peptide pools of WT, delta or omicron. (D) Relative fold‐change of AIM^+^ Th1 cell cross‐recognition of delta and omicron to WT (HC = 23, PAD = 15 in delta and 16 in omicron) and relative fold‐change of cTfh cell cross‐recognition of delta and omicron to WT (HC = 20, PAD = 16). (E) Representative gating of CD8^+^ AIM^+^ (CD69^+^4‐1BB^+^) T cell responses to spike peptide pools. (F) Fold‐change of AIM^+^ CD8^+^ T cell cross‐recognition of delta and omicron to WT (HC = 20, PAD = 16). Data are expressed as median with interquartile range. Significance of fold‐change decreases for each variant was evaluated by Wilcoxon‐signed rank *t*‐test compared with a hypothetical median of 1. Data are expression as median with interquartile range. ns, not significant.

CD4^+^ memory T cell recognition of delta and omicron by the AIM assay showed no significant decrease in both PAD and HC (Figure 3A,B). The frequencies of Th1 and cTfh cells that responded to delta and omicron spike peptide pools were similar in PAD and HC (Figure 3C,D). Consistent with CD4^+^ T cells, no significant decreases were observed in CD8^+^ T cells in recognition of delta and omicron spikes (Figure 3E,F). At the individual level, a decrease exceeding 10‐fold (0.1 by fold‐change) was not observed in any of the donors from both groups except 1 PAD in recognition to omicron BA.1 (Figure 3). Moreover, memory compartments within CD4^+^ and CD8^+^ populations were similar in cross‐recognition (Figures S7 and S8). Together, these results demonstrate that inactivated vaccine induced‐T cell cross‐recognition of delta and omicron are largely preserved in both PAD and HC.

### 3.5 Spike‐specific T cells cross‐recognize delta and omicron variants

**FIGURE 4:**
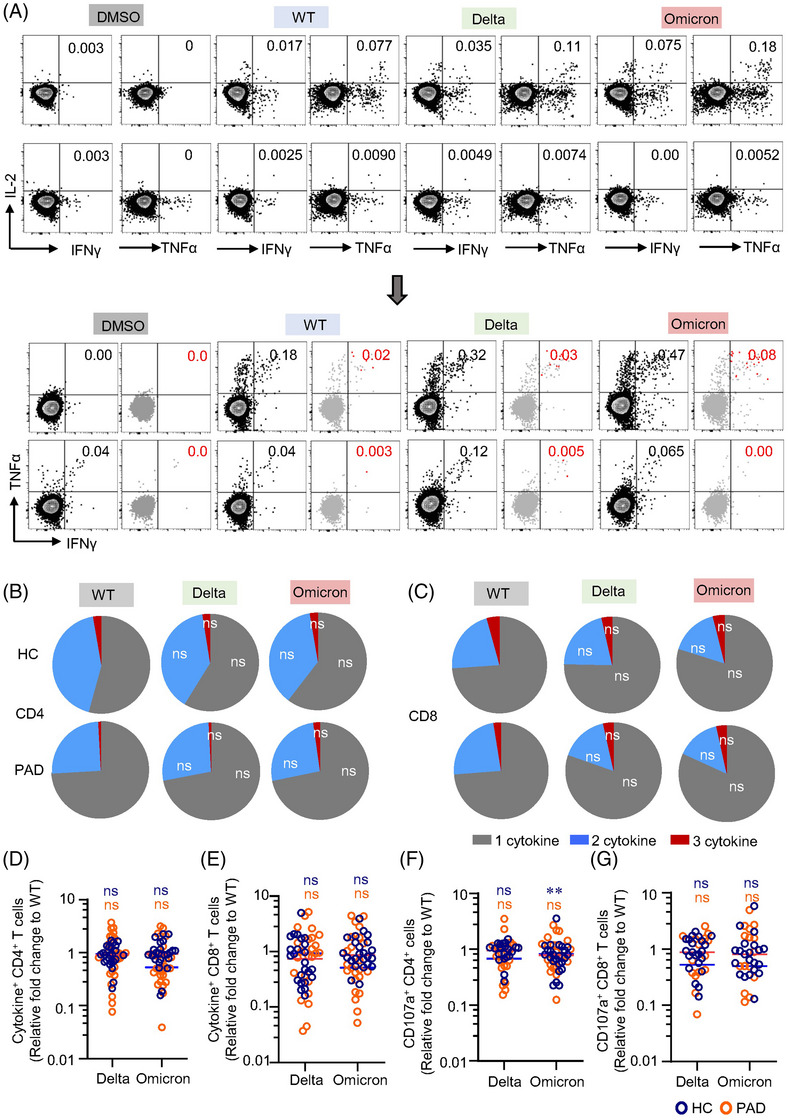
Impacts of variant‐associated mutations to inactivated vaccine induced spike‐specific T cell cytokine response. T cell reactivity to variants was assessed by intracellular cytokine staining (ICS) and cytotoxic T cells after stimulation with spike peptide pools of wild‐type (WT), delta and omicron BA.1. T cell reactivity to variants was relative to that of WT. T cells producing at least one of the three cytokines (IL‐2, TNFα and IFNγ) were defined as cytokine^+^ T cells. Gating strategy was performed as in Figure S2. (A) Representative flow cytometric plots depicting cytokine^+^ CD4^+^ or CD8^+^ T cells. (B, C) Cytokine profile of CD4^+^ and CD8^+^ T cell responses to peptide pools. (D) Relative fold‐change of cytokine^+^ CD4^+^ T cell cross‐recognition of delta and omicron to WT (HC = 24, PAD = 26). (E) Relative fold‐change of cytokine^+^ CD8^+^ T cell cross‐recognition of delta and omicron to WT (HC = 24, PAD = 25). (F) Relative fold‐change of CD4^+^ cytotoxic T cell cross‐recognition of delta and omicron to WT (HC = 24, PAD = 22). (G) Relative fold‐change of CD8^+^ cytotoxic T cell cross‐recognition of delta and omicron to WT (HC = 23, PAD = 18 in delta and 19 in omicron). Data are expressed as median with interquartile range. Significance of fold‐change decreases for each variant was evaluated by Wilcoxon‐signed rank *t*‐test compared with a hypothetical median of 1. Data are expression as median with interquartile range. ns, not significant, **p < .01

Antigen‐specific memory T cells associated cytokine response is essential for viral clearance.^42^, ^43^ We thus applied ICS to further assess the capability of cytokine response to delta and omicron in PAD and HC. Both CD4^+^ and CD8^+^ T cell responses to spike peptides derived from the 3 SARS‐CoV‐2 strains were polyfunctional (Figure 4A, Figure S9). The presence of combined IL‐2‐, TNFα‐ and IFNγ‐producing T cells suggests the capability of these memory cells in clearing SARS‐CoV‐2 virus during recall response. T cell functional profile in terms of cytokine production was similar between WT and VOCs in both PAD and HC (Figure 4B,C). Spike‐specific cytokine responses to delta or omicron were not affected compared to WT (Figure 4D,E). Overall, for delta variant, the median fold‐change was 0.90 (interquartile range 0.67–1.25) for HC and 0.93 (interquartile range 0.43–1.92) for PAD in CD4^+^ T cells, and 0.74 (interquartile range 0.32–1.64) for HC and 0.92 (interquartile range 0.23–1.73) for PAD in CD8^+^ T cells. As for omicron variant, the median fold‐change was 0.95 (interquartile range 0.74–1.15) for HC and 0.53 (interquartile range 0.37–0.98) for PAD in CD4^+^ T cells, and 0.86 (interquartile range 0.62–1.47) for HC and 0.51 (interquartile range 0.25–1.82) for PAD in CD8^+^ T cells. Looking into individuals, 1 PAD in cytokine^+^ CD4^+^ T cell responses and 2 PAD in cytokine^+^ CD8^+^ T cell responses showed decreases exceeding 10‐fold. In the HC group, no more than 10‐fold decreases were observed. Moreover, no significant decreases were detected in delta and omicron BA.1 variants in CD4^+^ and CD8^+^ CTL cells, except that CD4^+^ CTL for omicron BA.1 were observed to decrease in HC (Figure 4F,G, Figure S10). These results confirmed that polyfunctionality of spike‐specific T cells are preserved in response to delta and omicron in both PAD and HC.

### 3.6 **A third**‐**dose boosts CD4^+^ T cell responses in PAD to a greater magnitude**

**FIGURE 5:**
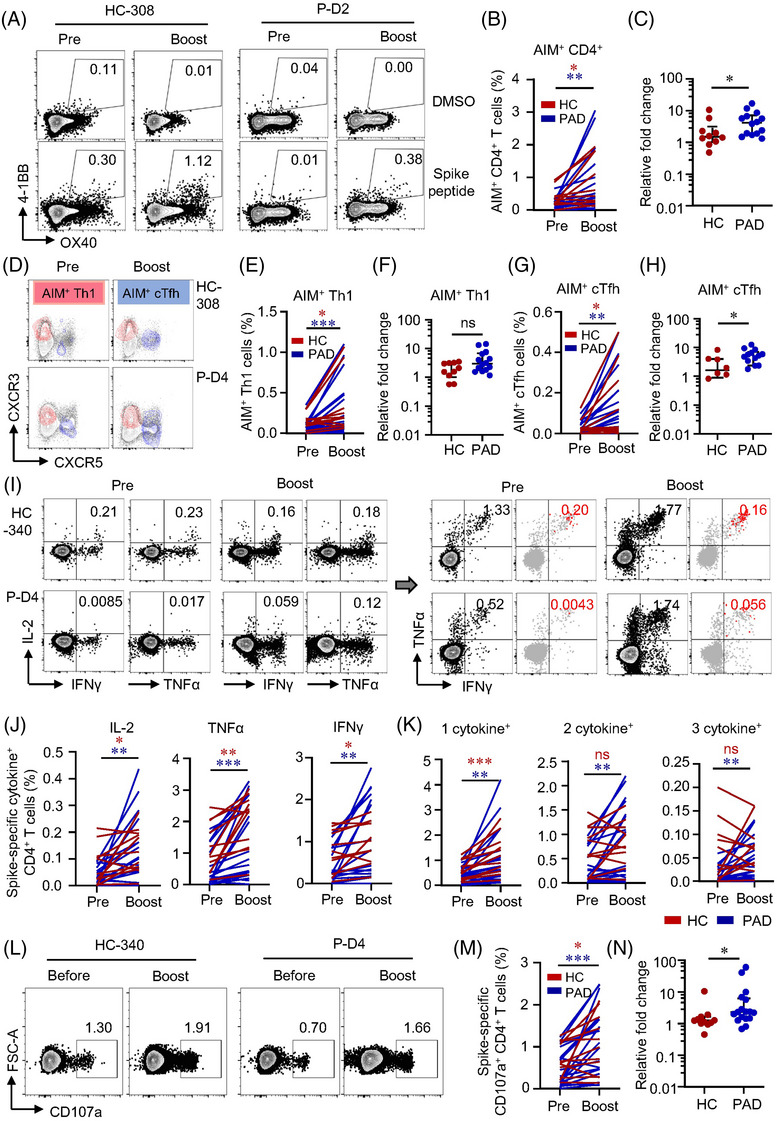
A third dose of inactivated vaccine boosts spike‐specific CD4^+^ T cell responses in patients with autoimmune diseases (PAD) to a greater extent. CD4^+^ T cell responses were assessed in PAD or healthy controls (HC) who received a third dose of inactivated vaccine 6 month after the prime vaccination by activation induced marker (AIM) and intracellular cytokine staining (ICS). (A) Representative flow cytometric plots showing AIM^+^ CD4^+^ T cells before and after the third vaccination. (B) Frequencies of spike‐specific AIM^+^ CD4^+^ T cells before and after the third dose of vaccine in PAD and HC. (C) Relative fold‐change of spike‐specific AIM^+^ CD4^+^ T cells by the third dose of vaccination (HC = 10, PAD = 14). (D) Representative flow cytometry plots depicting AIM^+^ Th1 and cTfh cells in PAD or HC. (E) Frequency of spike‐specific CD4^+^ Th1 cells (HC = 11, PAD = 18) and relative fold‐change of spike‐specific AIM^+^ CD4^+^ Th1 cells (HC = 10, PAD = 14) by the third dose (F). (G) Frequency of spike‐specific AIM^+^ CD4^+^ cTfh cells in PAD or HC before and after the third dose of vaccination (HC = 11, PAD = 18). (H) Relative fold‐change of AIM^+^ CD4^+^ cTfh cells before and after the third dose (HC = 8, PAD = 14). (I) Representative flow cytometric plots depicting the gating of cytokine^+^ CD4^+^ T cells. (J) Frequency of spike‐specific CD4^+^ cytokine^+^ (IL‐2, TNFα and IFNγ) T cells before and after third‐dose vaccine in PAD or HC (HC = 11, PAD = 18). (K) Comparisons of CD4^+^ T cells producing 1 cytokine, 2 cytokine and 3 cytokines between PAD or HC before and after third‐dose vaccine (HC = 11, PAD = 18). (L) Spike‐specific cytotoxic CD4^+^ T cells were defined by the expression of CD107a and representative flow cytometric plots were shown. (M) Frequencies of spike‐specific CD107a^+^ CD4^+^ T cells in PAD or HC before and after third‐dose vaccine (HC = 11, PAD = 18). (N) Relative fold‐change of spike‐specific cytotoxic CD4^+^ T by the third dose between HC and PAD (HC = 11, PAD = 17). In the fold‐change analysis, only donors with positive CD4^+^ T cell responses were included. Data are expressed as median with interquartile range. *p < .05, **p < .01 and ***p < .001 by paired Wilcoxon test in panels B, E, G, J, K, M and Mann–Whitney test in panels C, F, H, N. ns: not significant.

With the highly conserved but lower level of T cell responses induced by two‐dose of inactivated vaccine, boost effects of a third inactivated vaccine in PAD and HC remained to be explored. We found that general memory T cell compartments were unaffected (Figure S11). Spike‐specific AIM^+^ CD4^+^ T cells were positive in 14 out of 18 PAD and 10 out of 11 HC (Table S9) 6 months after the second dose, suggesting the long‐lasting of T cell responses in the vaccinees. Moreover, AIM^+^ and memory T cell pool were expanded in PAD and HC effectively by the third dose (Figure 5A,B, Figure S12). Interestingly, our data revealed that relative expansion of AIM^+^ CD4^+^ T cells were significantly greater in PAD than that in HC (Figure 5C). Frequencies of AIM^+^ Th1 and cTfh cells were also notably boosted by the third dose of vaccine in PAD and HC, with AIM^+^ cTfh expanded in a larger extent in PAD (Figure 5D–5H). The expansion of AIM^+^ Th1 cells was similar between PAD and HC (Figure 5F). In ICS analysis, we found that intracellular cytokine production in response to spike peptide was detected in most of the PAD and HC (Table S9). The production of IL‐2, TNFα and IFNγ were boosted by the third dose of vaccine in both PAD and HC (Figure 5I,J, Figure S13A). Also, the expansion of multifunctional CD4^+^ T cells expressing 2–3 cytokines was observed in PAD (Figure 5K). In addition, CD4^+^ CTL was expanded by a boost vaccine both in PAD and HC. Relative expansion of spike‐specific CD4^+^ CTL was consistently higher in PAD compared to HC (Figure 5L–N, Figure S13B). These data reveal that inactivated vaccine‐elicited CD4^+^ T cell responses are long‐lasting and could be boosted to a greater magnitude by a third dose in PAD (Figure S14), suggesting that PAD may benefit more from the boost dose.

### 3.7 Two‐dose **vaccine‐induced CD4^+^ T cells correlate with CD8^+^ T cell responses to a third dose**

**FIGURE 6:**
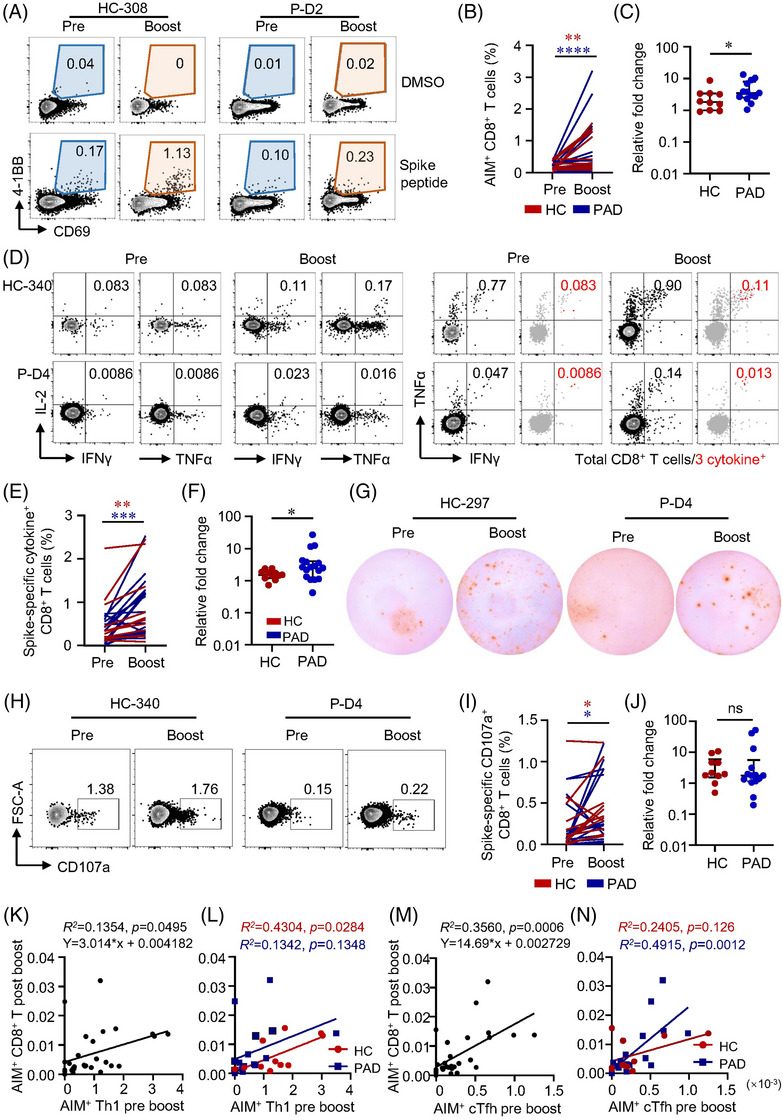
Spike‐specific CD4^+^ T cells correlate with CD8^+^ T cell responses by a third dose of inactivated vaccine. CD8^+^ T cell responses after a third dose of inactivated vaccine 6 month after the prime vaccination was evaluated using wild‐type (WT) spike peptide pool. (A) Activation induced marker (AIM^+^) CD8^+^ T cells in patients with autoimmune diseases (PAD) or healthy controls (HC) were measured by flow cytometry. Representative flow cytometric plots were shown. (B) Frequency of spike‐specific AIM^+^ CD8^+^ T cells before and after third dose in PAD or HC. (C) Comparison of fold‐change of spike‐specific AIM^+^ CD8^+^ T cells relative to that before third dose (HC = 10, PAD = 13 in fold‐change calculation). (D) Representative flow cytometric plots showing the gating of cytokine^+^ CD8^+^ T cells. (E) Frequency of spike‐specific cytokine^+^ CD8^+^ T cells before and after third‐dose vaccine in HC and PAD. (F) Comparison of fold‐change of spike‐specific cytokine^+^ CD8^+^ T cells relative to that before third dose (HC = 11, PAD = 17 in fold‐change calculation). (G) T cell responses to a third dose of vaccine were measured by IFNγ ELISpot. Representative images of IFNγ ELISpot wells before and after third dose in PAD and HC (HC = 6, PAD = 10). (H) Representative plots depicting CD107a expression in CD8^+^ T cells. (I, J) Frequency of spike‐specific cytotoxic CD8^+^ T cells and relative fold‐change before and after third dose (HC = 10, PAD = 14 in fold‐change calculation). (K–N) Correlation between the frequencies of AIM^+^ Th1 and cTfh cells before boost shot and frequency of AIM^+^ CD8^+^ T cells after boost shot respectively. In the fold‐change analysis, only donors with positive CD4^+^ T cell responses were included. Data are expressed as median with interquartile range. *p < .05, **p < .01 and ***p < .001 by paired Wilcoxon test in panels B, E, I and Mann–Whitney test in panels C, F, J. Linear regression analysis was performed in panels K–N. ns: not significant.

Seventeen out of 18 PAD and all HC after the boost were positive for AIM^+^ CD8^+^ T cells (Table S9). Both AIM expression assay and ICS showed the third dose of vaccine boosted CD8^+^ T cell responses in PAD and HC, and to a greater extent in PAD (Figure 6A–F, Figure S15). Ex vivo IFNγ ELISpot confirmed that a third dose of vaccine boosted T cell responses both in PAD and HC (Figure 6G). CD107a^+^ spike‐specific CD8^+^ CTL was consistently expanded by the third dose of vaccine, with similar relative expansion in PAD and HC (Figure 6H–J). After the third dose, CD8^+^ T cell responses were boosted to a greater extent in PAD that no differences were observed between PAD and HC (Figure S16). CD4^+^ T cells are primed rapidly after vaccination whereas CD8^+^ T cells develop gradually.^32^ Preexisting Th1 cells enhance CD8^+^ T cell responses.^44^ Our data revealed that the level of AIM^+^ Th1 cells before the third dose clearly predicted AIM^+^ CD8^+^ T cell responses after the boost dose by linear regression analysis (Figure 6K,L). Tfh cells provide help for optimal germinal centre and antibody responses.^45^ The frequency of AIM^+^ cTfh cells before the third dose was also closely correlated with AIM^+^ CD8^+^ T cell responses post‐boost (Figure 6 M,N), suggesting that humoral and cellular responses to the inactivated vaccine may not be completely separated and independent. In consistent with previous study,^46^ our data revealed that spike‐specific CD4^+^ T_SCM_ before boosted could predict CD8^+^ AIM^+^ T cells after boost, while CD4^+^ T_SCM_ could predict CD4^+^ AIM^+^ T cell responses only in the HC group (Figure S17). These data highlight the importance of boost vaccination in expanding CD8^+^ T cell responses in both groups. Moreover, these data also suggest that pre‐existence of specific CD4^+^ T cells may predict cellular response following boost vaccination.

### 3.8 **A third**‐**dose expands T cells cross‐recogni**z**e** d**elta and** o**micron variants**

**FIGURE 7:**
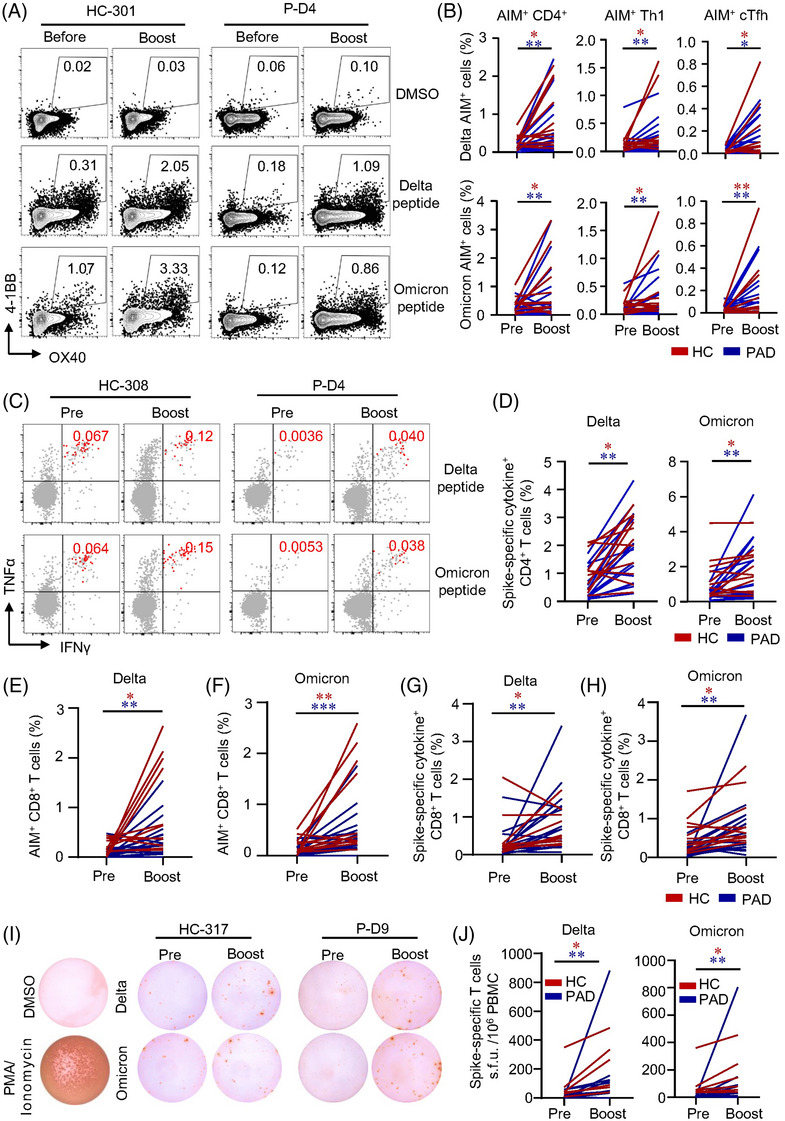
A third dose of inactivated vaccine expands spike‐specific CD4^+^ and CD8^+^ T cells cross‐recognize delta and omicron. T cell cross‐recognition to delta and omicron was assessed in healthy controls (HC) and patients with autoimmune diseases (PAD) who received a third dose by activation induced marker (AIM) and intracellular cytokine staining (ICS). (A) Representative flow cytometric plots depicting AIM^+^ CD4^+^ T cells. (B) Percentages of AIM^+^ CD4^+^ T cells were compared between pre‐ and post‐boost shot in PAD or HC. (C, D) Frequencies of spike‐specific AIM^+^ Th1 and cTfh cells before and after third vaccination in PAD or HC, respectively. (C) Representative flow cytometric plots showing cytokine^+^ CD4^+^ T cells before and after third dose. (D) Frequency of CD4^+^ cytokine^+^ (IL‐2, TNFα and IFNγ) T cell cross‐recognition of delta and omicron before and after third‐dose vaccine in PAD or HC. (E, F) Percentages of AIM^+^ CD8^+^ T cell cross‐recognition of delta and omicron were compared between pre‐ and post‐boost shot in PAD or HC, respectively. (G, H) Frequency of CD8^+^ cytokine^+^ (IL‐2, TNFα and IFNγ) T cell cross‐recognition of delta and omicron before and after third‐dose vaccine in PAD or HC. (I, J) T cell recognition of delta and omicron spikes were measured by IFNγ ELISpot. Representative images of ELISpot wells were shown. The numbers of IFNγ‐producing T cells were quantified by calculating the s.f.u. and summarised in (J) (HC = 6, PAD = 10). HC = 11, PAD = 16 in delta experiment and HC = 11, PAD = 17 in omicron experiment. Data are expressed as median with interquartile range. *p < .05 and **p < .01 by paired Wilcoxon test.

We found that delta and omicron spike‐responded CD4^+^ T cells and CD8^+^ T cells were found in majority of PAD and HC even 6 months after the second vaccination (Table S9). A third dose expanded AIM^+^ CD4^+^ T cells in PAD and HC (Figure 7A,B). AIM^+^ Th1 as well as AIM^+^ cTfh cells that responded to delta and omicron BA.1 peptide pools were induced significantly after the boost (Figure 7A,B). Moreover, ICS analysis revealed that these spike‐specific CD4^+^ T cells produced multiple cytokines including IL‐2, TNFα and IFNγ in response to delta and omicron spike peptides (Figure 7C,D). Importantly, no significant drop was observed in memory CD4^+^ T cell recognition of delta and omicron BA.1 in both PAD and HC (Figure S18). We found similar results in CD8^+^ T cells that a boost vaccine enhanced CD8^+^ T cell responses to delta and omicron BA.1 by AIM assay and ICS (Figure 7E–7H, Figure S19), which was further validated by ex vivo IFNγ ELISpot (Figure 7I,J). It is worth noted that CD107a^+^ CD4^+^ and CD8^+^ CTL were also expanded in PAD and HC significantly (Figures S18 and S19). Together, these data demonstrate that a third dose of inactivated vaccine enhances T cell responses to delta and omicron variants.

## 4 DISCUSSION

Here, we investigated T cell memory and their recognition of delta and omicron BA.1 variants after SARS‐CoV‐2 inactivated vaccination. Moreover, the effects of a third inactivated vaccine on WT, delta and omicron BA.1 were also studied. A robust and highly conserved T cell response to delta and omicron BA.1 variants was observed under two‐dose inactivated vaccine in both PAD and HC, however with a lower magnitude in PAD compared to HC. These data are consistent with studies on other vaccines including mRNA and viral vector‐based vaccines.^40^, ^47^, ^48^ Moreover, spike‐specific T cell immunity was greatly boosted by a third dose against WT, delta and omicron BA.1 strains in both PAD and HC. Surprisingly, the boost effect was greater in PAD, bringing similar levels in PAD and HC, demonstrating the importance of the boost dose for these patients.

Neutralising antibodies produced after vaccination are indispensable in preventing SARS‐CoV‐2 infection.^49^, ^50^ Although sera from individuals with infection history of SARS‐CoV‐2 variants show cross‐neutralization against each other, the continuing evolution of VOCs has affected the neutralising activities from vaccination or nature infection substantially.^51^, ^52^ Real‐world data have demonstrated that vaccine efficacy against omicron is substantially reduced in terms of infection prevention.^16^, ^53^ While vaccinated individuals are effectively protected from severe diseases and death.^54^ Early SARS‐CoV‐2‐specific IFNγ‐secreting T cells induction predicted milder symptom and helped viral clearance.^55^ For the broad range of spike‐specific T cell receptors, T cell responses could recognize multiple epitopes in VOCs disregard of mutated regions. Thus, T cell recognition of VOCs is relative conserved.^40^, ^48^ Vaccine‐induced specific T cell responses are unaffected by VOCs according to previous researches^56^, ^57^ and are largely preserved against diverse variants including delta and omicron.^48^, ^58^ In line with these studies, our data confirmed that SARS‐CoV‐2 inactivated vaccines induce robust T cell responses cross‐recognizing delta and omicron effectively.

Intracellular cytokine analysis revealed that inactivated vaccine‐induced T cell responses produce multiple cytokines including IL‐2, TNFα and IFNγ, suggesting the polyfunctionality of these antigen‐specific T cells. Importantly, cytokine response in spike‐specific T cells was preserved against delta and omicron BA.1 variants. Similar results have been reported in mRNA vaccine‐induced T cell responses.^59^ We also found spike‐specific CD107a^+^ CTL in these inactivated vaccine recipients. In line with the cytokine response, cytotoxic phenotype of the spike‐specific T cell cross‐recognized delta and omicron BA.1 spike protein. Given the important role of T cells for viral clearance, cellular immunity is important in preventing severe disease and fatal outcomes from viral infection. Our data revealed that inactivated vaccine induced‐T cells against strains of WT, delta and omicron BA.1 were detectable in both HC and PAD 6 months after the second dose, suggesting long‐lasting and functionally preserved T cell responses. However, spike‐specific T cell responses were impaired in PAD, pointing to the importance of a boost dose for PAD.

As has been noted that immunity against SARS‐CoV‐2 wanes over time after two‐dose of vaccination.^60^, ^61^, ^62^ A boost dose recalled SARS‐CoV‐2 specific responses rapidly and significantly.^30^, ^63^ T cell responses were lower in convalescents compared to 2‐time BNT162b2 vaccinated individuals when against omicron,^64^ highlighting the extra benefits of boost dose over nature immunity. Our previous study reported that a third inactivated vaccine expanded specific T cell responses against WT strain notably.^30^ Consistently, data in this study showed that spike‐specific T cells were expanded effectively in both PAD and HC after boost. Importantly, a third‐ dose expanded‐T cell cross‐recognition to delta and omicron variants was similar to WT strain. Similar results on inactivated vaccine were reported recently.^65^, ^66^ Surprisingly, the relative fold‐change of spike‐specific T cell responses boosted by the third dose was noted significantly greater in PAD compared to HC. The level of T cell responses was similar between PAD and HC after the boost. These results were confirmed by the real‐world data from Hongkong that two‐dose BNT162b2 had higher effectiveness in protecting against severe disease and death compared to CoronaVac during the omicron current recently. However, CoronaVac recipients benefited relative more from a third dose that three doses of either vaccine offered similar protection against severe outcomes,^54^ suggesting that boost vaccine could pose relative greater boost effects in previous low responders, older individuals and patients under immune suppression in particular.

Humoral and cellular responses waned faster in patients with immune mediated inflammatory diseases as shown in a previous study.^67^ Patients receiving immunosuppression agents showed impaired humoral and T cell responses to BNT162b2 mRNA or ChAdOx1 nCoV‐19 vaccines.^25^ Consistent with these studies, T cell responses and immune memory were detected lower in PAD compared to HC in this study. When comparing drug used rate between T cell responders and non‐responders, we found a significant higher hydroxychloroquine utilised rate in T cell responders according to ELISpot (Table S6–S8). As T cell responses to two‐dose of inactivated vaccine are low in PAD, a third dose is encouraged in these patients. Recently, data showed that T cell responses were induced in IBD and in immune‐mediated inflammatory diseases at levels similar to that of healthy individuals under SARS‐CoV‐2 mRNA vaccine platform.^28^, ^29^, ^68^ The cellular response was sustained even higher for up to half a year in IBD patients treated with TNF inhibitor.^29^ Thus, vaccination efficacies might be impacted by diseases activities, type and dosage of immune‐mediating drugs, combination therapy and disease type.

In summary, our data reveal that two‐dose of inactivated vaccine induces robust and durable T cell responses that could cross‐recognize delta and omicron variants in both PAD and HC. Spike‐specific T cells were lower in PAD after two‐dose, while a third dose enhances spike‐specific T cell responses to WT, delta and omicron BA.1 variants in PAD to a greater magnitude, demonstrating the importance of a boost dose for PAD. Importantly, the polyfunctionality of vaccine‐induced memory T cells is preserved in terms of cytokine and cytotoxic responses. Data presented in this study expand our understanding of the facture of T cell responses to inactivation vaccines in both PAD and HC, which could have potential important indications for vaccination strategies.

## 5 LIMITATION OF THIS STUDY

The participants recruited in this study were not from a cohort and samples were not collected and analysed longitudinally. The scope of the findings in this study is still limited. Larger sample size and cohort study will be valuable to extend our knowledge in understanding T cell recognition of VOCs elicited by SARS‐CoV‐2 inactivated vaccines. In addition, we did not analyse vaccine‐induced T cell responses to the omicron subvariants separately. Future studies are needed to further characterised T cell responses to the omicron subvariants. More, it is not clear whether these findings are clinically relevant. Finally, the long‐lasting of these memory T cells will require follow‐up studies.

## CONFLICT OF INTEREST

The authors declare that there is no conflict of interest that could be perceived as prejudicing the impartiality of the research reported.

## Supporting information


**TABLE S1** Participant characteristics in cohort 1
**TABLE S2** Participant characteristics in cohort 2
**TABLE S3** Antibody used in the study
**TABLE S4** Peptides used in the study
**TABLE S5** ELISpot reagents
**TABLE S6** CD4^+^ T cell response stratified by AIM^+^ T cells in cohort 1
**TABLE 7** CD8^+^ T cell response stratified by AIM^+^ T cell in cohort 1
**TABLE 8** T cell response stratified by IFNγ ELISpot in cohort 1
**TABLE 9** Summarisation of T cell response pre‐ and post‐third dose.
**FIGURE S1** Gating strategy for T cell analysis
**FIGURE S2** Gating strategies for T cell cytokine analysis
**FIGURE S3** T cell phenotypes in patients with autoimmune disease and healthy controls
**FIGURE S4** Spike‐specific T cell response in patient with autoimmune disease and healthy controls to two‐dose of inactivated vaccine
**FIGURE S5** Spike‐specific memory T cell phenotypes in patients with autoimmune disease and healthy controls in cohort 1
**FIGURE S6** Spike‐specific memory T cell phenotypes in patients with autoimmune disease and healthy controls in cohort 2
**FIGURE S7** Impacts of variant‐associated mutations on spike‐specific CD4^+^ T cell memory phenotype
**FIGURE S8** Impacts of variant‐associated mutations on spike‐specific CD8^+^ T cell memory phenotype
**FIGURE S**9 CD8^+^ T cell cytokine responses to delta and omicron in patients with autoimmune disease and healthy controls
**FIGURE S10** Two‐dose of inactivated vaccine elicits spike‐specific cytotoxic T cells cross‐recognize with delta and omicron in patients with autoimmune disease and healthy controls
**FIGURE S11** T cell subsets in patients with autoimmune disease and healthy controls before and after a third dose of inactivated vaccination
**FIGURE S12** A third dose of inactivated vaccine expands spike‐specific CD4^+^ T cell memory in patients with autoimmune disease and healthy controls
**FIGURE S13** Quality control plots for a third dose of inactivated vaccine boosts spike‐specific CD4^+^ T cell responses
**FIGURE S14** Spike‐specific CD4^+^ T cell responses after the third dose of vaccine in patients with autoimmune diseases and HC
**FIGURE S15** Quality control plots for a third dose of inactivated vaccine boosts spike‐specific CD8^+^ T cell responses
**FIGURE S16** Spike‐specific CD8^+^ T cell responses after the third dose of vaccine in patients with autoimmune diseases and HC
**FIGURE S17** Correlation between the frequencies of CD4^+^ AIM^+^ T_SCM_ cells before boost shot and frequency AIM^+^ T cells after boost shot
**FIGURE S18** Spike‐specific CD4^+^ T cell responses to Delta and Omicron by a third dose of vaccine
**FIGURE S19** Spike‐specific CD8^+^ T cell responses to Delta and Omicron by a third dose of vaccine
