# Supplementary material for: Impacts of delta and omicron variants on inactivated SARS‐CoV‐2 vaccine‐induced T cell responses in patients with autoimmune diseases and healthy controls
Source: Clin Transl Med. 2023 Jan 13;13(1):e1171. doi: 10.1002/ctm2.1171 (PMC9839884; doi:10.1002/ctm2.1171)
Supplement: Supplementary file 1 — TABLE S1 Participant characteristics in cohort 1 TABLE S2 Participant characteristics in cohort 2 TABLE S3 Antibody used in the study TABLE S4 Peptides used in the study TABLE S5 ELISpot reagents TABLE S6 CD4+ T cell response stratified by AIM+ T cells in cohort 1 TABLE 7 CD8+ T cell response stratified by AIM+ T cell in cohort 1 TABLE 8 T cell response stratified by IFNγ ELISpot in cohort 1 TABLE 9 Summarisation of T cell response pre‐ and post‐third dose. FIGURE S1 Gating strategy for T cell analysis FIGURE S2 Gating strategies for T cell cytokine analysis FIGURE S3 T cell phenotypes in patients with autoimmune disease and healthy controls FIGURE S4 Spike‐specific T cell response in patient with autoimmune disease and healthy controls to two‐dose of inactivated vaccine FIGURE S5 Spike‐specific memory T cell phenotypes in patients with autoimmune disease and healthy controls in cohort 1 FIGURE S6 Spike‐specific memory T cell phenotypes in patients with autoimmune disease and healthy controls in cohort 2 FIGURE S7 Impacts of variant‐associated mutations on spike‐specific CD4+ T cell memory phenotype FIGURE S8 Impacts of variant‐associated mutations on spike‐specific CD8+ T cell memory phenotype FIGURE S9 CD8+ T cell cytokine responses to delta and omicron in patients with autoimmune disease and healthy controls FIGURE S10 Two‐dose of inactivated vaccine elicits spike‐specific cytotoxic T cells cross‐recognize with delta and omicron in patients with autoimmune disease and healthy controls FIGURE S11 T cell subsets in patients with autoimmune disease and healthy controls before and after a third dose of inactivated vaccination FIGURE S12 A third dose of inactivated vaccine expands spike‐specific CD4+ T cell memory in patients with autoimmune disease and healthy controls FIGURE S13 Quality control plots for a third dose of inactivated vaccine boosts spike‐specific CD4+ T cell responses FIGURE S14 Spike‐specific CD4+ T cell responses after the third dose of vaccine in p [file CTM2-13-e1171-s001.docx]

**Supplementary data**

**Supplementary Table 1.** Participant characteristics in cohort 1.

| **Cohort 1** | | **PAD** | | **HC** | ***p*** |
| --- | --- | --- | --- | --- | --- |
| Participants (no.) | | 56 | | 25 |  |
| Age (mean±SD) | | 38.3 (14.4) | | 37.5 (9.3) | 0.80 |
| Gender: female (no, %) | | 42 (75.0) | | 18 (72.0) | 0.79 |
| Body mass index (mean±SD) | | 21.5 (2.6) | | 21.1 (2.2) | 0.48 |
| Diagnosis no. (%) | |  | |  |  |
| Systemic lupus erythematosus | | 29 (51.8) | |  |  |
| Rheumatoid arthritis | | 11 (19.6) | |  |  |
| Ankylosing spondylitis | | 9 (16.1) | |  |  |
| Primary Sjogren’s syndrome | | 7 (12.5) | |  |  |
| Days after second dose (mean±SD) | | 115.9 (29.7) | | 120.1 (25.0) | 0.54 |
| Adverse events to vaccination no. (%) | | 6 (10.7) | | 4 (16.0) |  |
| Injection site symptoms | Induration | 4 (7.1) | | 3 (12.00) |  |
|  | Swollen | 1 (1.8) | | 1 (4.0) |  |
| Systematic symptoms | Fatigue | 1 (1.8) | | 1 (4.0) |  |
|  | Fever | 2 (3.6) | | 0 (0.0) |  |
|  | Myalgia | 2 (3.6) | | 1 (4.0) |  |
| Respiratory symptoms | Sore throat | 1 (1.8) | | 1 (4.0) |  |
|  | Running nose | 1 (1.8) | | 0 (0.0) |  |
|  | Cough | 1 (1.8) | | 1 (4.0) |  |
| Concomitant Medication no. (%) | |  | |  |  |
| Any csDMARDs | | 34 (60.71) | |  |  |
| Methotrexate | | 12 (21.42) | |  |  |
| Mycophenolate Mofetil | | 16 (28.57) | |  |  |
| Cyclosporine A | | 5 (8.93) | |  |  |
| Leflunomide | | 1 (1.79) | |  |  |
| Hydroxychloroquine | | 37 (66.07) | |  |  |
| Sulfasalazine | | 1 (1.79) | |  |  |
| Prednisone | | 22 (39.29) |  | |  |
| bDMARDs | | 14 (25.00) |  | |  |
| Disease activity scores (mean±SD) | | Pre/Post Vaccination | | |  |
| SLEDAI | | 3.4/3.3 |  | | >0.999 |
| DAS28 | | 2.9/2.7 |  | | 0.29 |
| ESSDAI | | 1.1/0.8 |  | | 0.50 |
| BASDAI | | 0.2/0.24 |  | | >0.999 |

Abbreviations: csDMARDs, conventional synthetic disease-modifying antirheumatic drugs; bDMARDs, biological DMARDs; SLEDAI, SLE disease activity index; DAS28, Disease Activity Score 28; ESSDAI, European League Against Rheumatism SS disease activity index; BASDAI, Bath Ankylosing Spondylitis Disease Activity Index. PAD, patients with autoimmune diseases; HC, healthy controls.

**Supplementary Table 2.** Participant characteristics in cohort 2.

| **Cohort 2** | | **PAD** | **HC** | | | ***p*** |
| --- | --- | --- | --- | --- | --- | --- |
| Participants (no.) | | 28 | | 24 | |  |
| Age (mean±SD) | | 41.5 (16.1) | | 37.6 (11.3) | | 0.32 |
| Gender: female (no., %) | | 23 (82.1) | | 21 (87.5) | | 0.91 |
| Body mass index (mean±SD) | | 21.4 (2.5) | | 20.8 (2.4) | | 0.29 |
| Diagnosis (no., %) | |  | |  | |  |
| Systemic lupus erythematosus | | 12 (42.9) | |  | |  |
| Rheumatoid arthritis | | 6 (21.4) | |  | |  |
| Ankylosing spondylitis | | 5 (17.9) | |  | |  |
| Primary Sjogren’s syndrome | | 5 (17.9) | |  | |  |
| Days after second dose (mean±SD) | | 206.2 (30.6) | | 204.6 (26.8) | | 0.93 |
| Adverse events to vaccination no. (%) | | 4 (14.3) | | 3 (12.5) |  | |
| Injection site symptoms | Induration | 3 (10.7) | | 2 (7.1) |  | |
|  | Swollen | 0 (0.0) | | 0 (0.0) |  | |
| Systematic symptoms | Fatigue | 1 (3.6) | | 1 (4.2) |  | |
|  | Fever | 0 (0.0) | | 0 (0.0) |  | |
|  | Myalgia | 1 (3.6) | | 0 (0.0) |  | |
| Respiratory symptoms | Sore throat | 0 (0.0) | | 1 (4.2) |  | |
|  | Running nose | 0 (0.0) | | 0 (0.0) |  | |
|  | Cough | 1 (3.6) | | 1 (4.2) |  | |
| Concomitant Medication no. (%) | |  | |  |  | |
| Any csDMARDs | | 17 (60.7) | |  |  | |
| Mycophenolate Mofetil | | 12 (42.9) | |  |  | |
| Hydroxychloroquine | | 10 (35.7) | |  |  | |
| Prednisone | | 15 (53.6) | |  |  | |
| bDMARDs | | 7 (25.0) | |  |  | |
| Disease activity scores (mean±SD) | | Pre-/post boost | |  |  | |
| SLEDAI | | 2.9/3.1 | |  | | >0.999 |
| DAS28 | | 3.0/3.0 | |  | | 0.88 |
| ESSDAI | | 0.15/0.18 | |  | | >0.999 |
| BASDAI | | 0.067/0.13 | |  | | - |

Abbreviations: csDMARDs, conventional synthetic disease-modifying antirheumatic drugs; bDMARDs, biological DMARDs; SLEDAI, SLE disease activity index; DAS28, Disease Activity Score 28; ESSDAI, European League Against Rheumatism SS disease activity index; BASDAI, Bath Ankylosing Spondylitis Disease Activity Index. PAD, patients with autoimmune disease; HC, healthy control.

**Supplementary Table 3.** Antibody used in the study.

| **Antigens** | **Fluorophores** | **Source** | **Clones** | **Identifier** |
| --- | --- | --- | --- | --- |
| CD3 | Pacific Blue | BioLegend | HIT3a | Cat# 300330 |
| CD4 | AF488 | BioLegend | OKT4 | Cat# 317420 |
| CD8 | PerCP | BioLegend | SK1 | Cat# 344708 |
| CD69 | APC | BioLegend | FN50 | Cat# 310910 |
| 4-1BB | PE | BioLegend | 4B4-1 | Cat# 309804 |
| OX40 | BV605 | BioLegend | ACT35 | Cat# 350028 |
| CCR7 | APC/Cyanine7 | BioLegend | G043H7 | Cat#353212 |
| CD45RA | BV650 | BioLegend | HI100 | Cat# 304136 |
| CXCR5 | BV711 | BioLegend | J252D4 | Cat# 356934 |
| CXCR3 | BV510 | BioLegend | G025H7 | Cat# 353726 |
| CD3 | FITC | Biolegend | HIT3a | Cat# 300306 |
| IFNγ | BV421 | BioLegend | 4S.B3 | Cat# 502532 |
| TNFα | APC/Cyanine7 | BioLegend | MAb11 | Cat# 502944 |
| IL-2 | BV650 | BioLegend | MQ1-17H12 | Cat#500334 |
| CCR7 | Pacific Blue | BioLegend | G043H7 | Cat# 353210 |
| CD45RA | AF 700 | BioLegend | HI100 | Cat# 304120 |
| CD107a | BV785 | BioLegend | H4A3 | Cat# 328644 |
| CD95 | PE/Cyanine5 | BioLegend | DX2 | Cat# 305610 |
| Zombie Red | / | BioLegend | / | Cat# 423110 |

**Supplementary Table 4.** Peptides used in the study.

| **Peptides** | **Source** | **Identifier** |
| --- | --- | --- |
| SARS-CoV-2 S defined peptide pool | MabTech | Cat# 3630-1 |
| SARS-CoV-2 Spike Glycoprotein-Crude | GenScript | Cat# RP30020 |
| SARS-CoV-2-Spike-B.1.617.2-Delta | GenScript | Cat# RP30033 |
| SARS-CoV-2 Spike-B.1.1.529-Omicron | GenScript | Cat# RP30121 |

**Supplementary Table 5.** ELISpot reagents.

| **Antibodies and reagents** | **Source** | **Identifier** |
| --- | --- | --- |
| Anti-human IFN-gamma mAb | MabTech | Cat# 3420-3-250 |
| Anti-human IFN-gamma mAb | MabTech | Cat# 3420-6-250 |
| Horseradish Peroxidase Avidin D | Vector | Cat# A-2004-5 |
| AEC coloring system | DAKEWE | Cat# 2030613 |
| Magi coating buffer | DAKEWE, | Cat# 2030111 |
| ELISpot PVDF plates, clear | MabTech | Cat# 3654-TP-10 |

**Supplementary Table 6.** CD4^+^ T cell response stratified by AIM^+^ T cells in cohort 1.

| **CD4^+^ T cell response** | **AIM (PAD)** | | ***p*** | **Medicine** | |
| --- | --- | --- | --- | --- | --- |
|  | **-** | **+** |  | **- (AIM %)** | **+ (AIM %)** |
| AIM^+^ (no.) | 9 | 47 |  |  |  |
| Age (mean) | 34.9 | 38.5 | 0.49 |  |  |
| Female (%) | 77.8 | 74.5 | >0.99 |  |  |
| BMI (mean) | 20.3 | 21.8 | 0.09 |  |  |
| Days after second dose (mean) | 121.2 | 114.9 | 0.57 |  |  |
| Concomitant Medication (%) |  |  |  |  |  |
| Any csDMARDs  Methotrexate  Mycophenolate Mofetil  Cyclosporine A  Hydroxychloroquine  Prednisone  NSAIDs  bDMARDs | 88.9 | 55.3 | 0.14 | 0.25% | 0.25% |
|  | 11.1 | 23.4 | 0.70 | 0.25% | 0.27% |
|  | 44.4 | 25.5 | 0.46 | 0.25% | 0.28% |
|  | 22.2 | 6.4 | 0.37 | 0.25% | 0.20% |
|  | 33.3 | 72.3 | 0.06 | 0.13% | 0.31%* |
|  | 55.6 | 36.2 | 0.47 | 0.25% | 0.23% |
|  | 11.1 | 6.4 | >0.99 | 0.25% | 0.19% |
|  | 11.1 | 27.7 | 0.53 | 0.27% | 0.22% |

*p<0.05 by Mann-Whitney test.

Abbreviations: AIM: Activation induced marker; PAD: patients with autoimmune diseases; csDMARDs, conventional synthetic disease-modifying antirheumatic drugs; bDMARDs, biological DMARDs.**Supplementary Table 7.** CD8^+^ T cell response stratified by AIM^+^ T cell in cohort 1.

| **CD8^+^ T cell response** | **AIM (PAD)** | | ***p*** | **Medicine** | |  |
| --- | --- | --- | --- | --- | --- | --- |
|  | **-** | **+** |  | **-**  **(AIM %)** | **+ (AIM %)** | |
| AIM^+^ (no.) | 22 | 34 |  |  |  | |
| Age (mean) | 36.0 | 39.2 | 0.41 |  |  | |
| Female/male (%) | 81.1 | 70.6 | 0.53 |  |  | |
| Body mass index (mean) | 21.1 | 21.9 | 0.20 |  |  | |
| Days after second vaccine (mean) | 109.8 | 119.9 | 0.22 |  |  | |
| Concomitant Medication (%) |  |  |  |  |  | |
| Any csDMARDs  Methotrexate  Mycophenolate Mofetil  Cyclosporine A  Hydroxychloroquine  Prednisone  NSAIDs  bDMARDs | 59.1 | 61.8 | 0.43 | 0.085% | 0.080% | |
|  | 9.1 | 29.4 | 0.14 | 0.070% | 0.17% | |
|  | 27.3 | 29.4 | >0.99 | 0.075% | 0.11% | |
|  | 18.2 | 2.9 | 0.14 | 0.10% | 0.00%^#^ | |
|  | 63.6 | 67.6 | 0.99 | 0.13% | 0.070% | |
|  | 50.0 | 32.4 | 0.30 | 0.11% | 0.035% | |
|  | 4.5 | 8.8 | 0.94 | 0.075% | 0.13% | |
|  | 27.3 | 23.5 | >0.99 | 0.08% | 0.14% | |

^#^ p=0.055 by Mann-Whitney test.

Abbreviations: AIM: Activation induced marker; PAD: patients with autoimmune diseases; csDMARDs, conventional synthetic disease-modifying antirheumatic drugs; bDMARDs, biological DMARDs; PAD, patients with autoimmune disease.

**Supplementary Table 8.** T cell response stratified by IFNγ ELISpot in cohort 1.

| **T Cell Response** | **ELISpot (PAD)** | | ***p*** | **Medicine (s.f.u/10^6^ PBMCs)** | |
| --- | --- | --- | --- | --- | --- |
|  | **-** | **+** |  | **-** | **+** |
| ELISpot (no.) | 18 | 38 |  |  |  |
| Age (mean) | 38.0 | 37.9 | 0.99 |  |  |
| Female (%) | 83.3 | 71.1 | 0.51 |  |  |
| Body mass index | 21.1 | 21.8 | 0.31 |  |  |
| Days after second dose (mean) | 107.8 | 119.8 | 0.16 |  |  |
| Concomitant Medication (%) |  |  |  |  |  |
| Any csDMARDs  Methotrexate  Mycophenolate Mofetil  Cyclosporine  Hydroxychloroquine  Prednisone  NSAIDs  bDMARDs | 66.7 | 57.9 | 0.61 | 80.0 | 32.5 |
|  | 16.7 | 23.7 | 0.80 | 35.0 | 42.5 |
|  | 27.8 | 28.9 | >0.99 | 42.5 | 35.0 |
|  | 16.7 | 5.3 | 0.37 | 45.0 | 0.0* |
|  | 44.4 | 76.3 | 0.04* | 20.0 | 85.0* |
|  | 44.4 | 36.8 | 0.80 | 35.0 | 42.5 |
|  | 5.6 | 7.9 | >0.99 | 35.0 | 77.5 |
|  | 38.9 | 18.4 | 0.19 | 45.0 | 22.5 |

*p<0.05 by Mann-Whitney test.

Abbreviations: PAD: patients with autoimmune diseases; s.f.u: spot-forming units csDMARDs, conventional synthetic disease-modifying antirheumatic drugs; bDMARDs, biological DMARDs; PAD, patients with autoimmune disease.

**Supplementary Table 9.** Summarization of T cell response pre- and post-third dose.

| **Measurements** | | **PAD (pre/post)** | | |  | **HC (pre/post)** | | |
| --- | --- | --- | --- | --- | --- | --- | --- | --- |
|  |  | **WT**  **(n=18)** | **Delta**  **(n=16)** | **Omicron**  **(n=17)** |  | **WT**  **(n=11)** | **Delta**  **(n=11)** | **Omicron**  **(n=11)** |
| **CD4^+^ AIM^+^ (n)** | 14/17 | | 15/15 | 12/16 |  | 10/11 | 10/11 | 10/11 |
| **CD8^+^ AIM^+^ (n)** | 13/17 | | 8/14 | 10/14 |  | 10/11 | 9/11 | 9/11 |
| **CD4 Cytokine (n)** |  | |  |  |  |  |  |  |
| IL-2 | 14/15 | | 11/15 | 8/13 |  | 8/10 | 8/9 | 7/10 |
| TNFα | 17/18 | | 14/15 | 15/15 |  | 11/11 | 10/11 | 11/11 |
| IFNγ | 14/17 | | 15/15 | 16/16 |  | 11/11 | 10/11 | 10/11 |
| At least one cytokine | 18/18 | | 16/16 | 17/17 |  | 11/11 | 11/11 | 11/11 |
| **CD8 Cytokine (n)** |  | |  |  |  |  |  |  |
| IL-2 | 8/10 | | 5/10 | 5/8 |  | 7/7 | 6/7 | 7/7 |
| TNFα | 14/17 | | 11/16 | 11/16 |  | 10/11 | 9/10 | 10/11 |
| IFNγ | 11/10 | | 10/12 | 11/12 |  | 9/10 | 6/10 | 7/8 |
| At least one cytokine | 15/18 | | 12/16 | 14/17 |  | 10/11 | 9/11 | 10/11 |
| **Cytotoxic T cells (n)** |  | |  |  |  |  |  |  |
| CD4^+^ | 17/18 | | 15/15 | 15/17 |  | 11/11 | 10/11 | 11/11 |
| CD8^+^ | 14/18 | | 11/15 | 12/15 |  | 10/11 | 10/11 | 9/10 |
| At least one | 16/18 | | 15/15 | 15/17 |  | 11/11 | 11/11 | 11/11 |

Abbreviations: PAD, patients with autoimmune disease; HC, healthy control. AIM: Activation induced marker


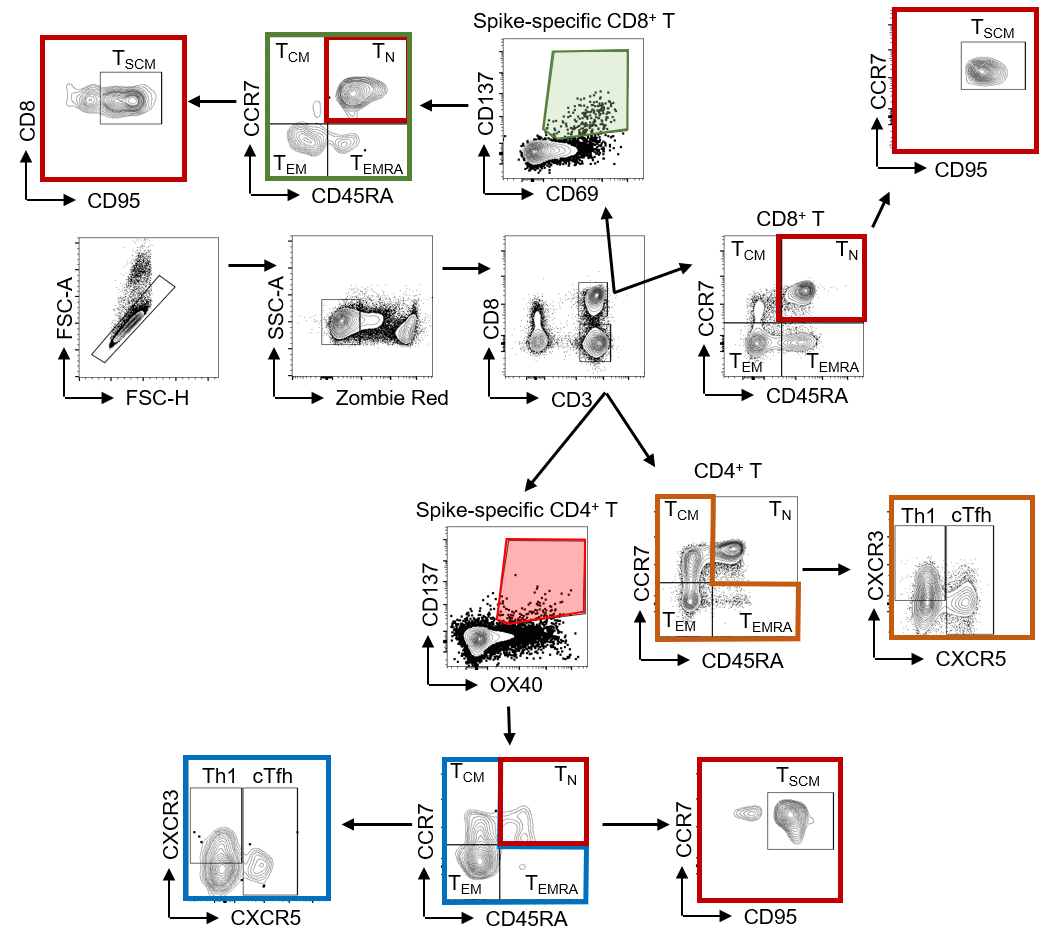


Supplementary Figure 1**. Gating strategy for T cell analysis.** Gates are shown sequentially for identifying AIM^+^ T cells, memory T cell subsets. T cell phenotyping: spike-specific AIM^+^ CD4^+^ T cells: OX40^+^4-1BB^+^, spike-specific AIM^+^ CD8^+^ T cells: CD69^+^4-1BB^+^, Th1 cells: CD4^+^CXCR3^+^CXCR5^-^, cTfh cells: CD4^+^CXCR5^+^, naïve T cells (T_N_): CD45RA^+^CCR7^+^, effector memory T cells (T_EM_): CD45RA^-^CCR7^-^, central memory T cells (T_CM_): CD45RA^-^CCR7^+^, terminally differentiated T cells (T_EMRA_): CD45RA^+^CCR7^-^. T_SCM_: CD45RA^+^CCR7^+^CD95^+^.


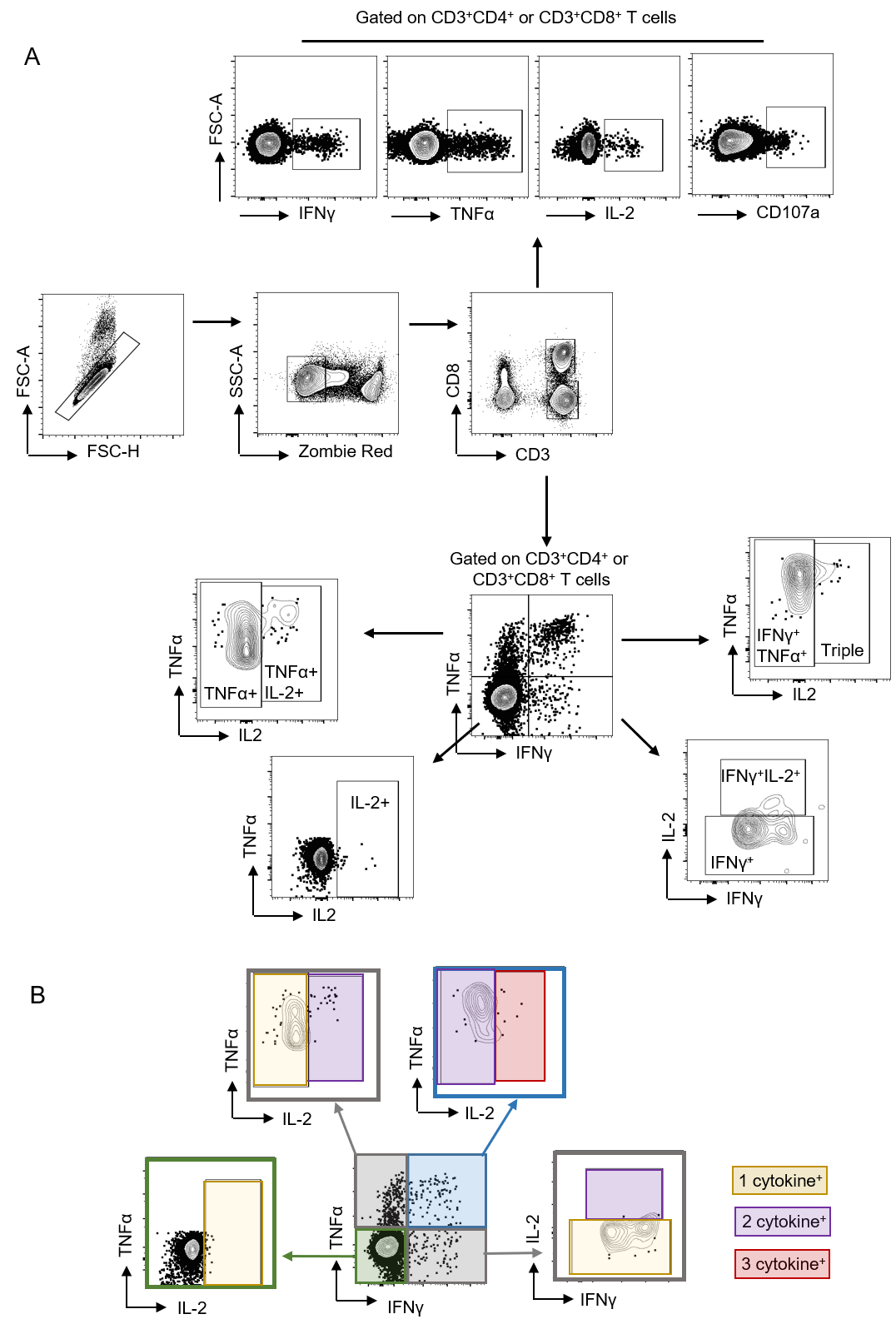


Supplementary Figure 2**. Gating strategies for T cell cytokine analysis. (A)** Gates are shown sequentially for cytokine expression analysis. (B) . Based on the kinds of cytokine produced, spike-specific CD4^+^ T cells were divided into 1 cytokine^+^ (producing anyone of IFNγ, TNFα and IL-2), 2 cytokines^+^ (producing any two of IFN-γ, TNF-α and IL-2) and 3 cytokines^+^ (producing all 3 cytokines) spike-specific CD4^+^ T cells.


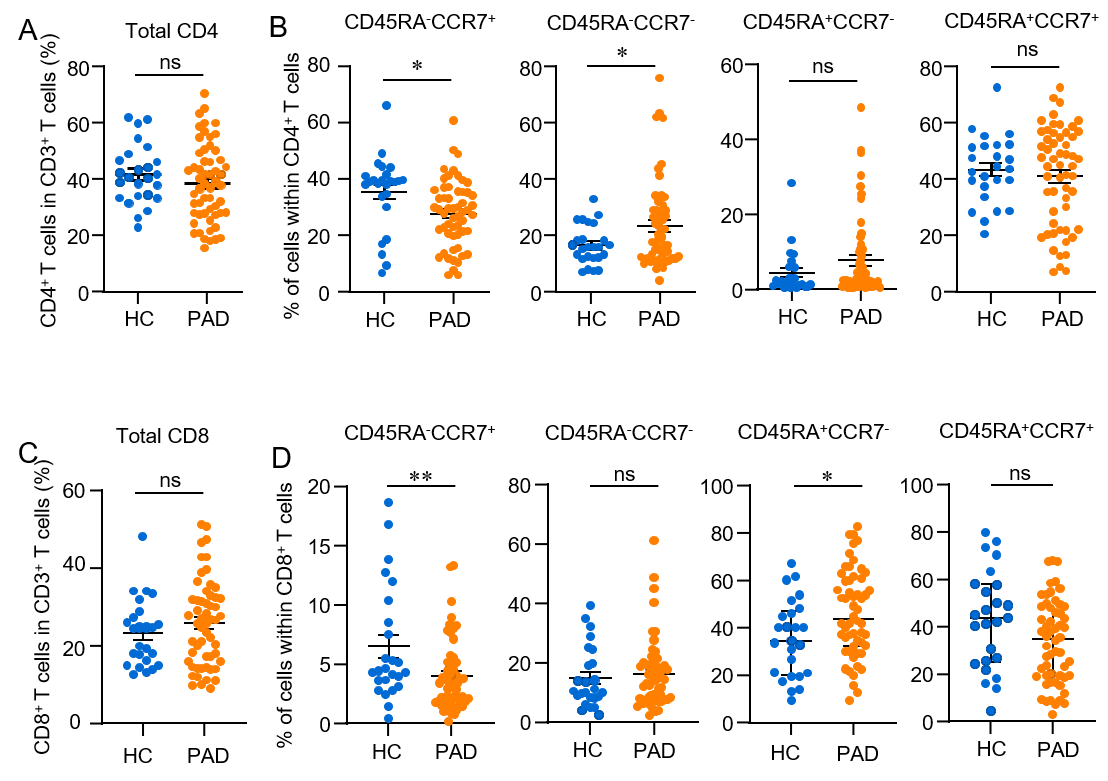


Supplementary Figure **3.** **T cell phenotypes in patients with autoimmune disease and healthy controls**. Blood samples were collected from patients with autoimmune diseases (PAD) or HC who finished 2-dose vaccine. (A, C) Proportions of total CD4^+^ or CD8^+^ T cell subsets gated in CD3^+^ lived T cells from patients and HC. (B, D) Proportions of memory T cell subsets (CD45RA^-^CCR7^+^, CD45RA^-^CCR7^-^, CD45RA^+^CCR7^-^, CD45RA^+^CCR7^+^) in CD4^+^ or CD8^+^ T cells from patients and HC. HC=25, PAD=56. Data are expression as median with interquartile range. *p<0.05, **p<0.01 by Mann-Whitney test. ns: not significant.


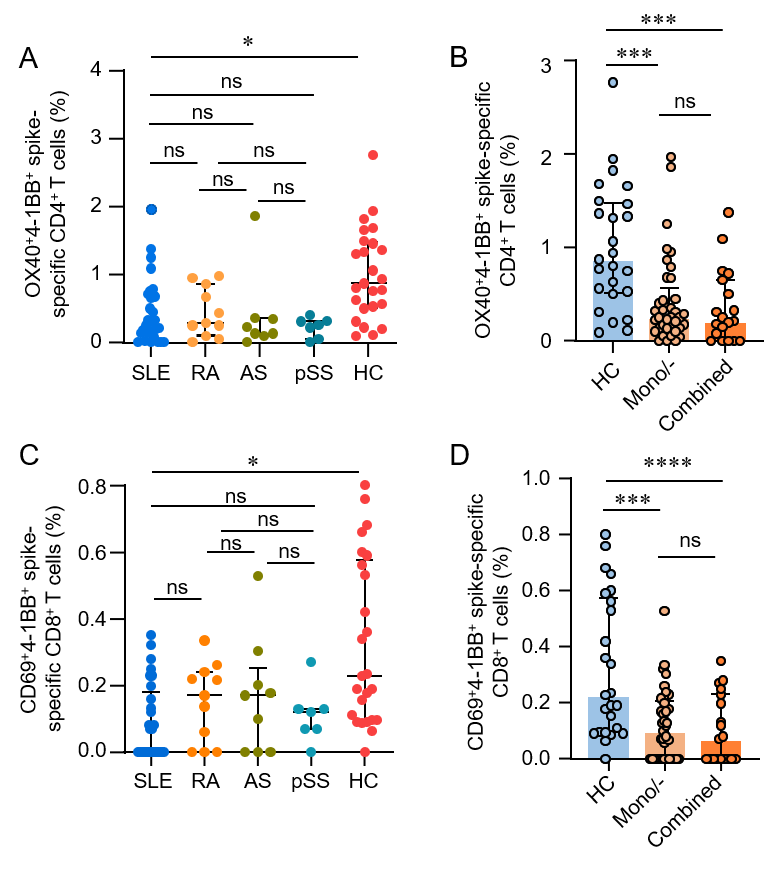


Supplementary Figure 4. **Spike-specific T cell response in patient with autoimmune disease and healthy controls to 2-dose of inactivated vaccine**. (A) Frequency of spike specific CD4^+^ T cell (OX40^+^ 4-1BB^+^) in patient and HC. (B) Frequency of spike specific CD4^+^ T cell (OX40^+^ 4-1BB^+^) in patient with monotherapy or combined therapy and HC. (C) Frequency of SARS-CoV-2 spike specific CD8^+^ T cell (CD69^+^ 4-1BB^+^) in patient and HC. (D) Frequency of SARS-CoV-2 spike specific CD8^+^ T cell (CD69^+^ 4-1BB^+^) in patient with monotherapy or combined therapy and HC. SLE=29, RA=11, AS=9, SS=7, HC=25. Monotherapy, no more than one kind of DMARDs and Glucocorticoid, Combined therapy, more than one kind of DMARDs and Glucocorticoid. Data are expression as median with interquartile range. Ordinary one-way ANOVA followed by multiple comparison correction. *p<0.05，ns: not significant. SLE: systemic lupus erythematosus, RA: rheumatoid arthritis, AS: ankylosing spondylitis, SS: primary sojgren’s syndrome.


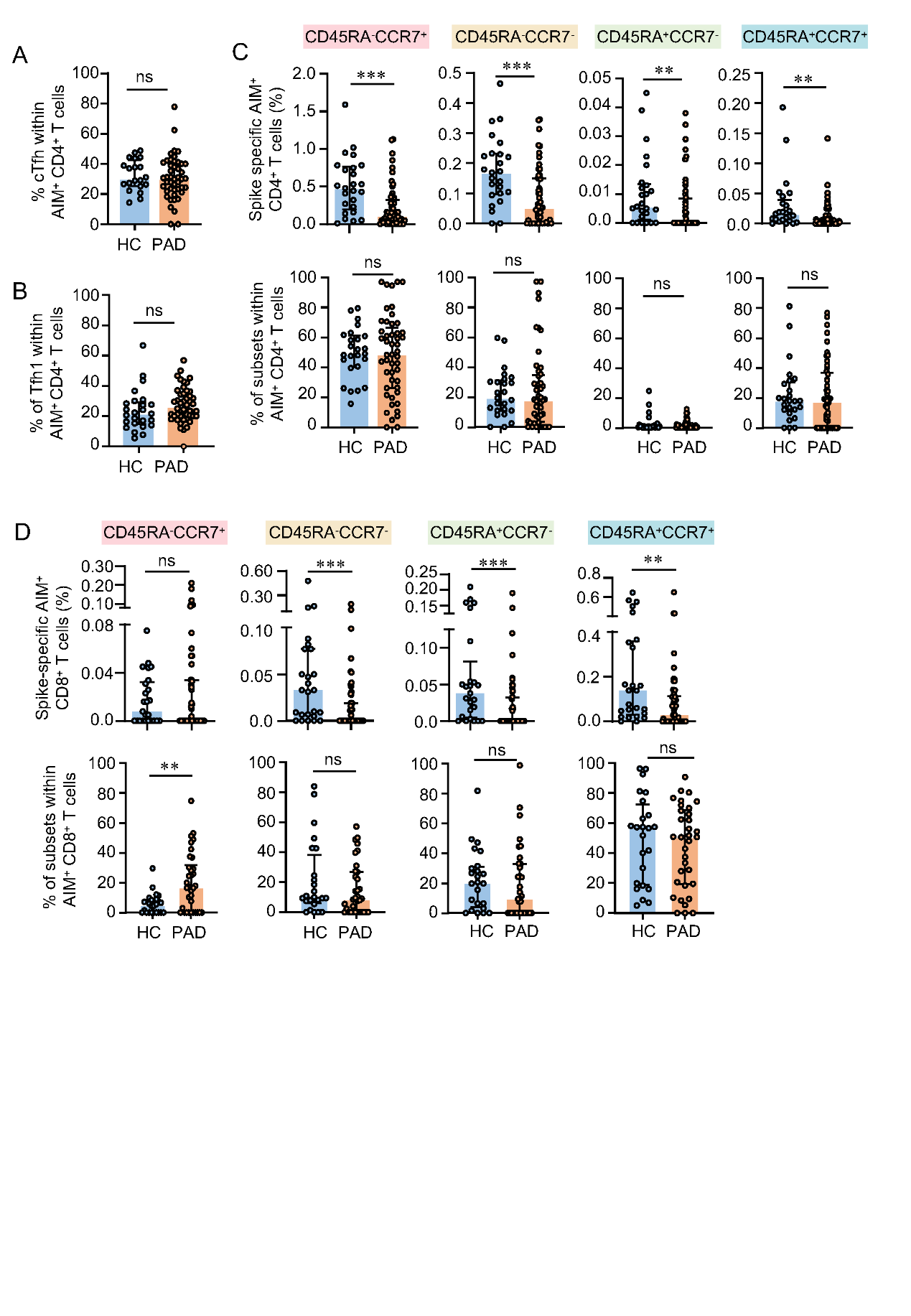


Supplementary Figure 5. **Spike-specific memory T cell phenotypes in patients with autoimmune disease and healthy controls in cohort 1**. Spike-specific memory T cell distribution was further analyzed for the expression of CD45RA, CCR7 and CD95 by flow cytometry. ). (A, B) Proportion of cTfh and Th1 AIM^+^ CD4^+^ cells in HC and PAD (PAD=56, HC=25). (C) Percentages and proportions of CD45RA^-^CCR7^+^, CD45RA^-^CCR7^-^, CD45RA^+^CCR7^-^, CD45RA^+^CCR7^+^ AIM^+^ CD4^+^ cells in HC and PAD (PAD=56, HC=25) (D) Percentages and proportions of CD45RA^-^CCR7^+^, CD45RA^-^CCR7^-^, CD45RA^+^CCR7^-^, CD45RA^+^CCR7^+^ AIM^+^ CD8^+^ cells in HC and PAD (PAD=56, HC=25). Data are expression as median with interquartile range. *p<0.05, **p<0.01, ***p<0.001 by Mann-Whitney test. ns: not significant.


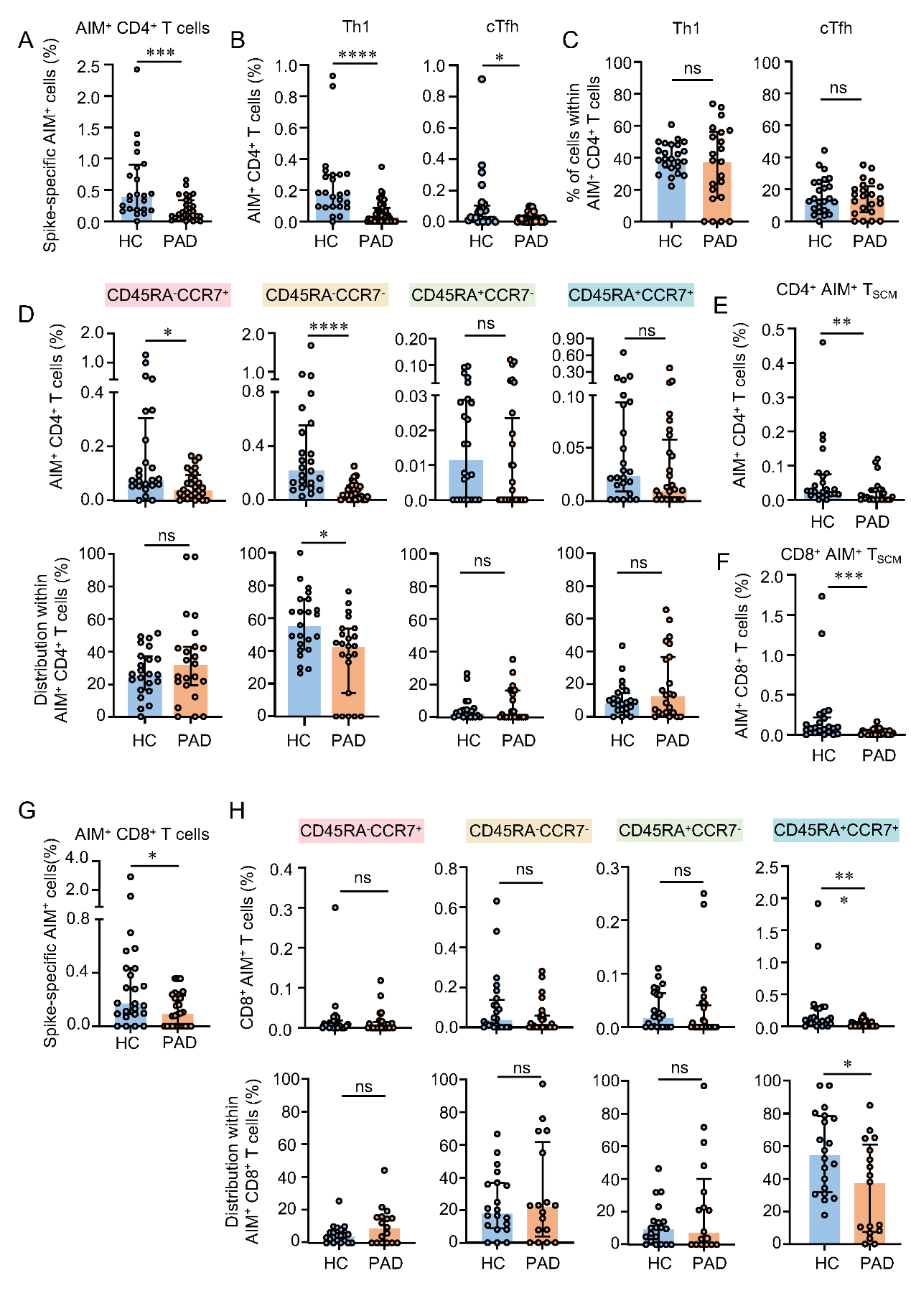


Supplementary Figure 6. **Spike-specific memory T cell phenotypes in patients with autoimmune disease and healthy controls in cohort 2.** Frequency and distribution of spike-specific memory T cell was analyzed by flow cytometry in cohort 2 pre-boost. (A) Percentages of spike-specific AIM^+^ (OX40^+^4-1BB^+^) CD4^+^ T cells as in PAD or HC. (B, C) Comparisons of CD4^+^ AIM^+^ Th1 and cTfh cells between HC and PAD respectively. (D) Percentages and proportions of CD45RA^-^CCR7^+^, CD45RA^-^CCR7^-^, CD45RA^+^CCR7^-^, CD45RA^+^CCR7^+^ AIM^+^ CD4^+^ cells in HC and PAD. (E) Percentages of CD45RA^+^CCR7^+^CD95^+^ AIM^+^ CD4^+^ cells (AIM^+^ CD4^+^ T_SCM_ cells) in PAD or HC. (F) Percentages of CD45RA^+^CCR7^+^CD95^+^ AIM^+^ CD8^+^ cells (AIM^+^ CD8^+^ T_SCM_ cells) in PAD or HC. (G) Percentages of spike-specific AIM^+^ (CD69^+^4-1BB^+^) CD8^+^ T cells as in PAD or HC. (H) Percentages and proportions of CD45RA^-^CCR7^+^, CD45RA^-^CCR7^-^, CD45RA^+^CCR7^-^, CD45RA^+^CCR7^+^ AIM^+^ CD8^+^ cells in HC and PAD. PAD=28, HC=24. Data are expression as median with interquartile range. *p<0.05, **p<0.01, ***p<0.001 by Mann-Whitney test. ns: not significant.


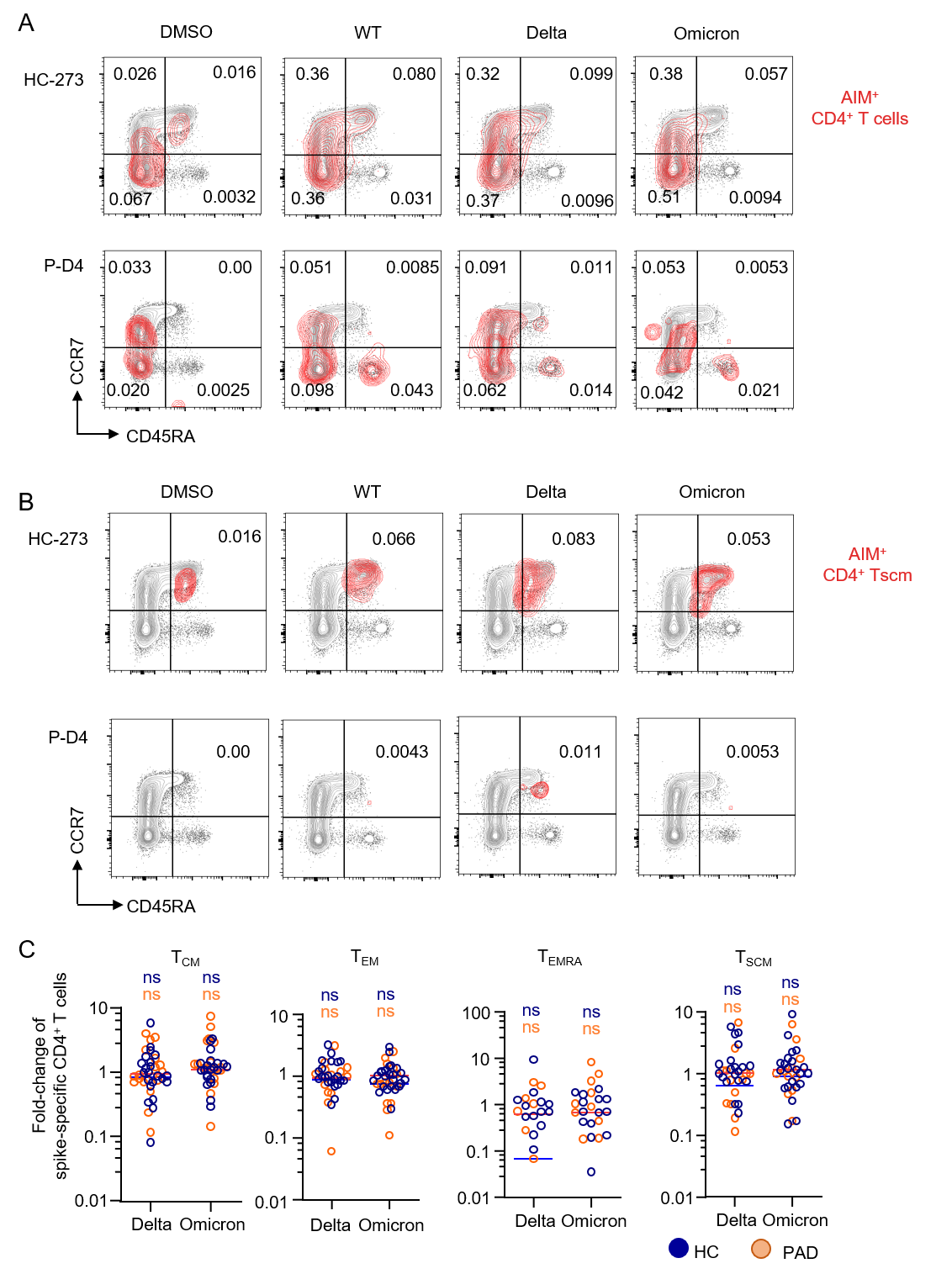


Supplementary Figure 7. **Impacts of variant-associated mutations on spike-specific CD4^+^ T cell memory phenotype.** CD4^+^ T cell response to variants of Delta and Omicron were assessed using spike peptide pools. The effects of Delta and Omicron variants were expressed as relative to WT. (A) Representative flow cytometric plots for the AIM^+^ CD4^+^ T cells to identify T cell memory subsets after the second dose of inactivated vaccine. Red: AIM^+^ CD4^+^ T cells, gray: total CD4^+^ T cells from the same donor. Upper row: HC donors, lower row: patient donors. (B) Representative flow cytometric plots for the expression of CD4^+^ T_SCM_ (CD4^+^CD45RA^+^CCR7^+^CD95^+^) cells in patient and HC donors. Red: CD4^+^ T_SCM_ cells, gray: total CD4^+^ T cells from the same donor. upper row: HC donors, lower row: patient donors. (C) Fold-change of AIM^+^ CD4^+^ T cell memory subsets for Delta and Omicron is relative to WT (each dot represents an independent sample). Significance of fold-change decreases for each variant was assessed by Wilcoxon signed rank T test compared with a hypothetical median of 1. ns, not significant.


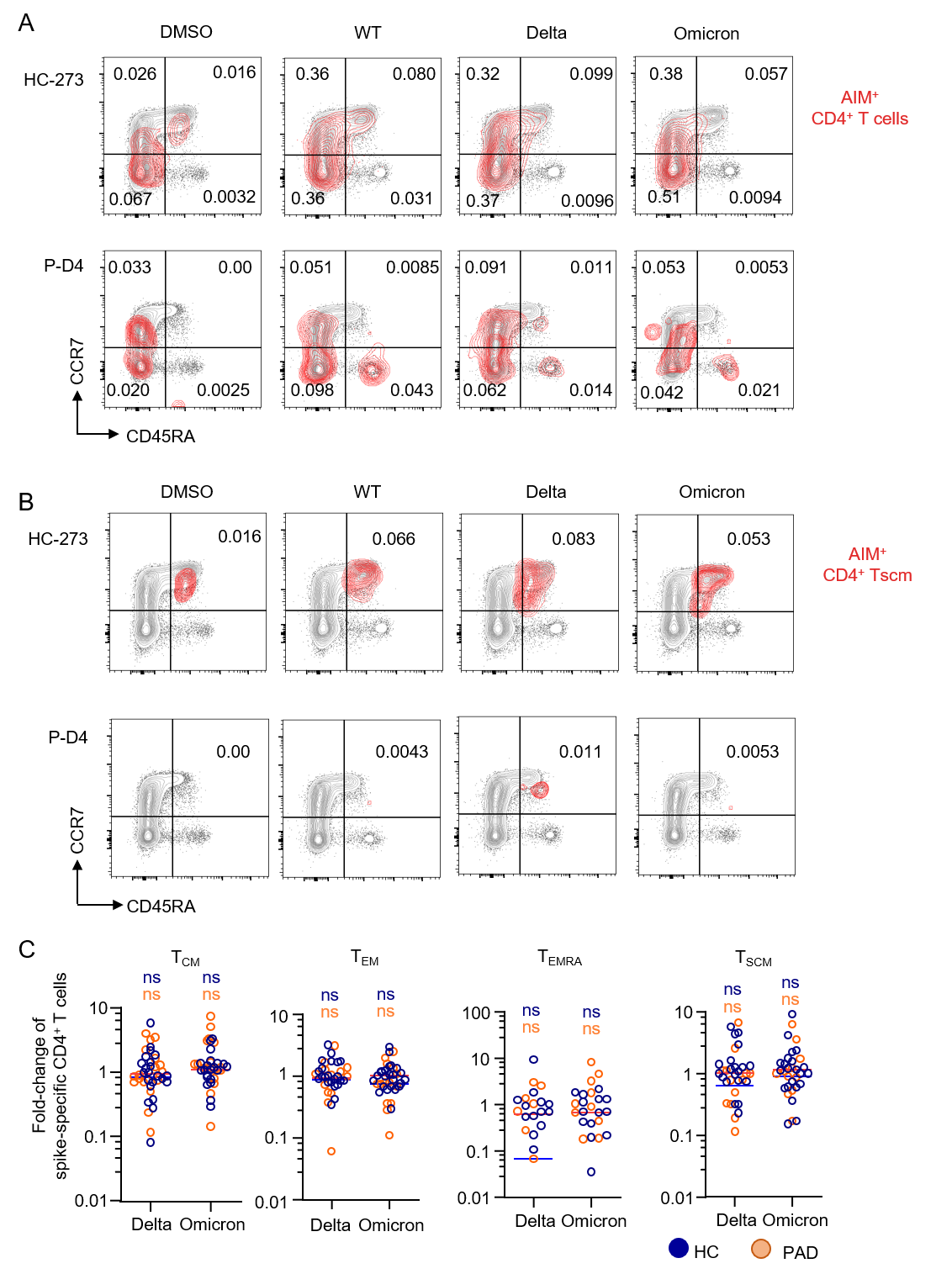


Supplementary Figure 8. **Impacts of variant-associated mutations on spike-specific CD8^+^ T cell memory phenotype.** CD8^+^ T cell response to variants of Delta and Omicron were assessed using spike peptide pools. The effects of Delta and Omicron variants were expressed as relative to WT. (A) Representative flow cytometric plots for the AIM^+^CD8^+^ T cells to identify T cell memory subsets after the second dose of inactivated vaccine. Red: AIM^+^CD8^+^ T cells, gray: total CD8^+^ T cells from the same donor. Upper row: HC donors, lower row: PAD donors. (B) Representative flow cytometric plots for the expression of CD8^+^ T_SCM_ (CD8^+^CD45RA^+^CCR7^+^CD95^+^) cells in PAD and HC donors. Red: CD8^+^ T_SCM_ cells, gray: total CD8^+^ T cells from the same donor. upper row: HC donors, lower row: PAD donors. (C) Fold-change of AIM^+^ CD8^+^ T cell memory subsets for Delta and Omicron is relative to WT in HC and PAD (each dot represented an independent sample). Significance of fold-change decreases for each variant was assessed by Wilcoxon signed rank T test compared with a hypothetical median of 1.


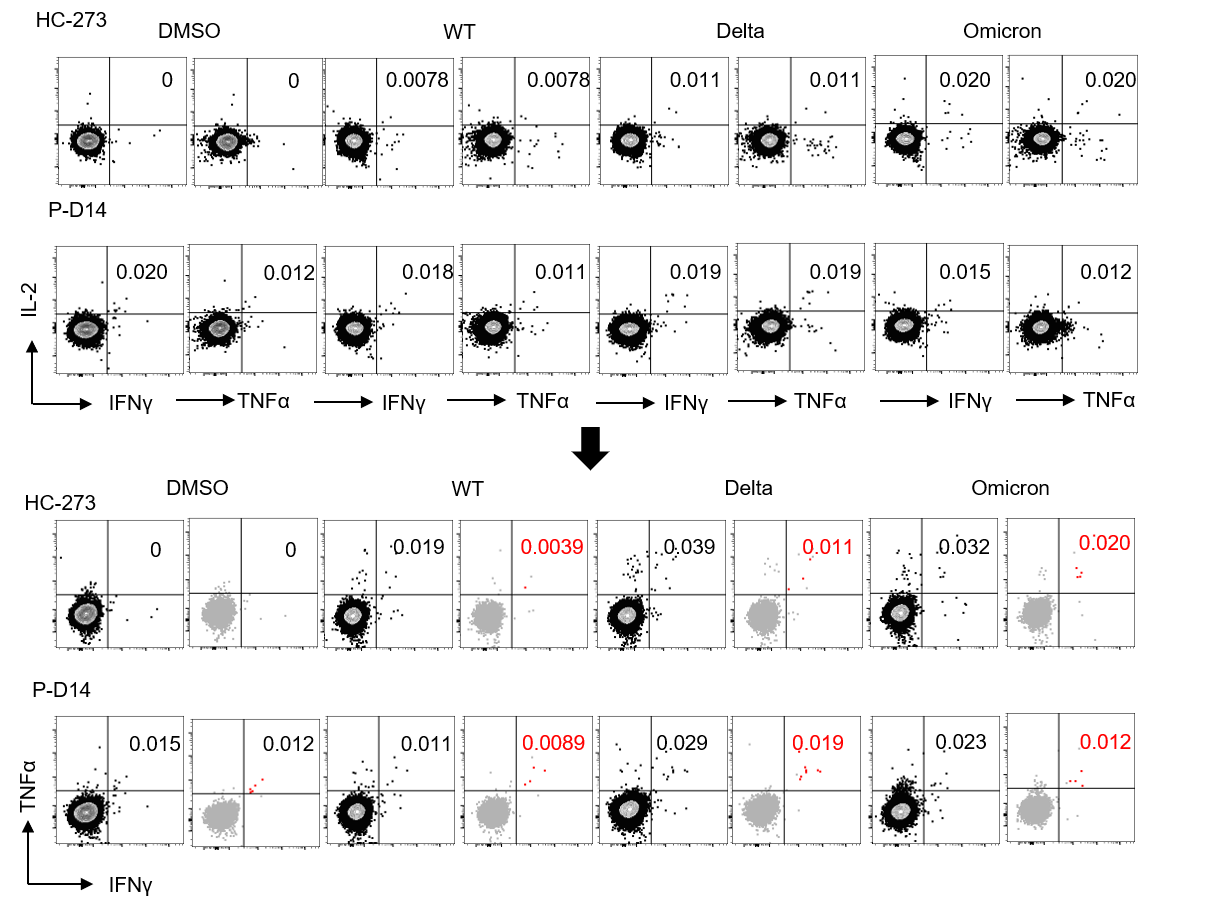


Supplementary Figure 9. **CD8^+^ T cell cytokine responses to Delta and Omicron in patients with autoimmune disease and healthy controls.** Representative flow cytometric plots depicting cytokine producing CD8^+^ T cells. The first and third row: HC donors, the second and fourth row: PAD donors. Red: TNFα^+^IFNγ^+^IL-2^+^ CD8^+^ T cells, gray: CD8^+^ T cells. Numbers indicate the frequency of cytokine^+^ cells falling within each gate.


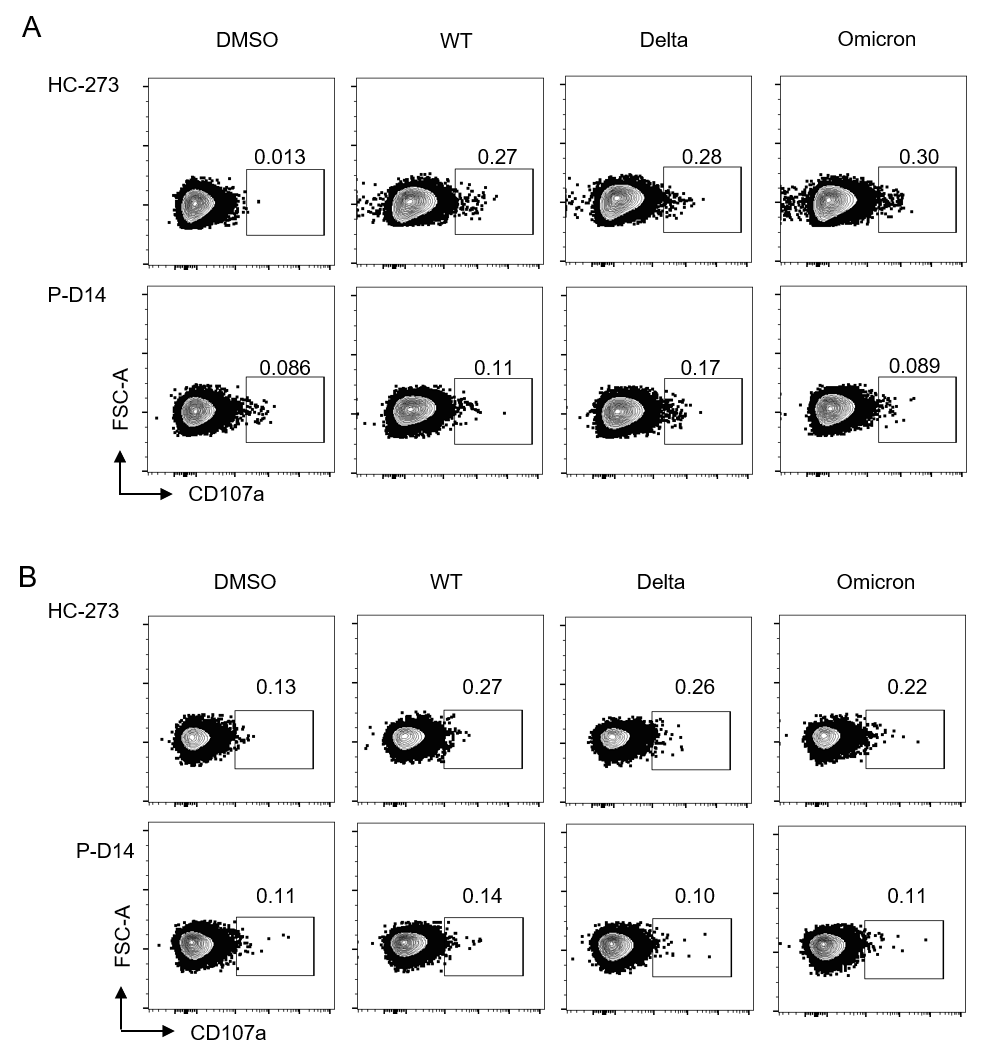


Supplementary Figure 10**. Two-dose of inactivated vaccine elicits spike-specific cytotoxic T cells cross recognize with Delta and Omicron in patients with autoimmune disease and healthy controls.** (A) Representative flow cytometric plots depicting spike-specific CD107a^+^ CD4^+^ T cells. (B) Representative flow cytometric plots depicting spike-specific CD107a^+^ CD8^+^ T cells. Top row: HC donors, bottom row: PAD donors.


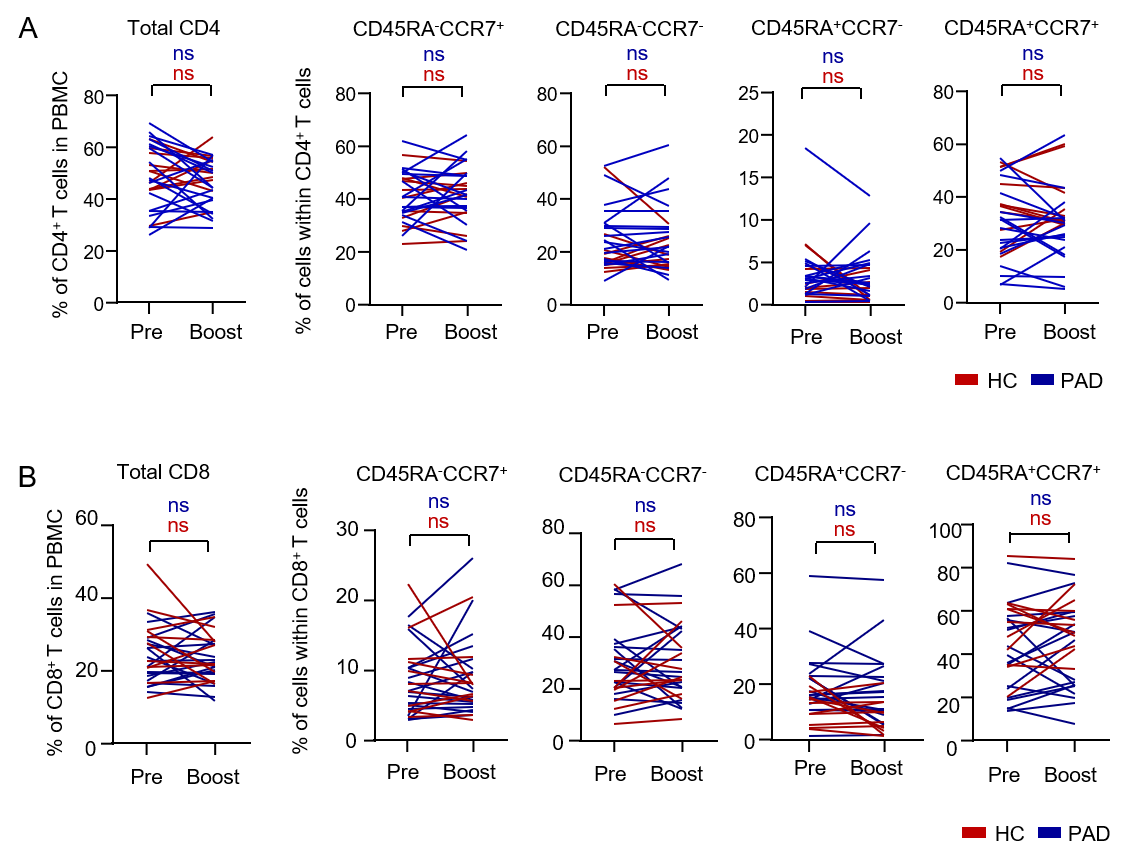


Supplementary Figure 11**. T cell subsets in patients with autoimmune disease and healthy controls before and after a third dose of inactivated vaccination.** (A) Proportions of total CD4^+^ T cells in PBMC lived cells and memory subsets from PAD or HC before and after the third dose. (B) Proportions of total CD8^+^ T cells in PBMC lived cells and memory subsets from PAD or HC before and after the third dose. HC=11, PAD=18. T_CM_: CD45RA^-^CCR7^+^, T_EM_: CD45RA^-^CCR7^-^, T_EMRA_: CD45RA^+^CCR7^-^, T_N_: CD45RA^+^CCR7^+^. Data are expression as median with interquartile range. ns: not significant by paired Wilcoxon test.


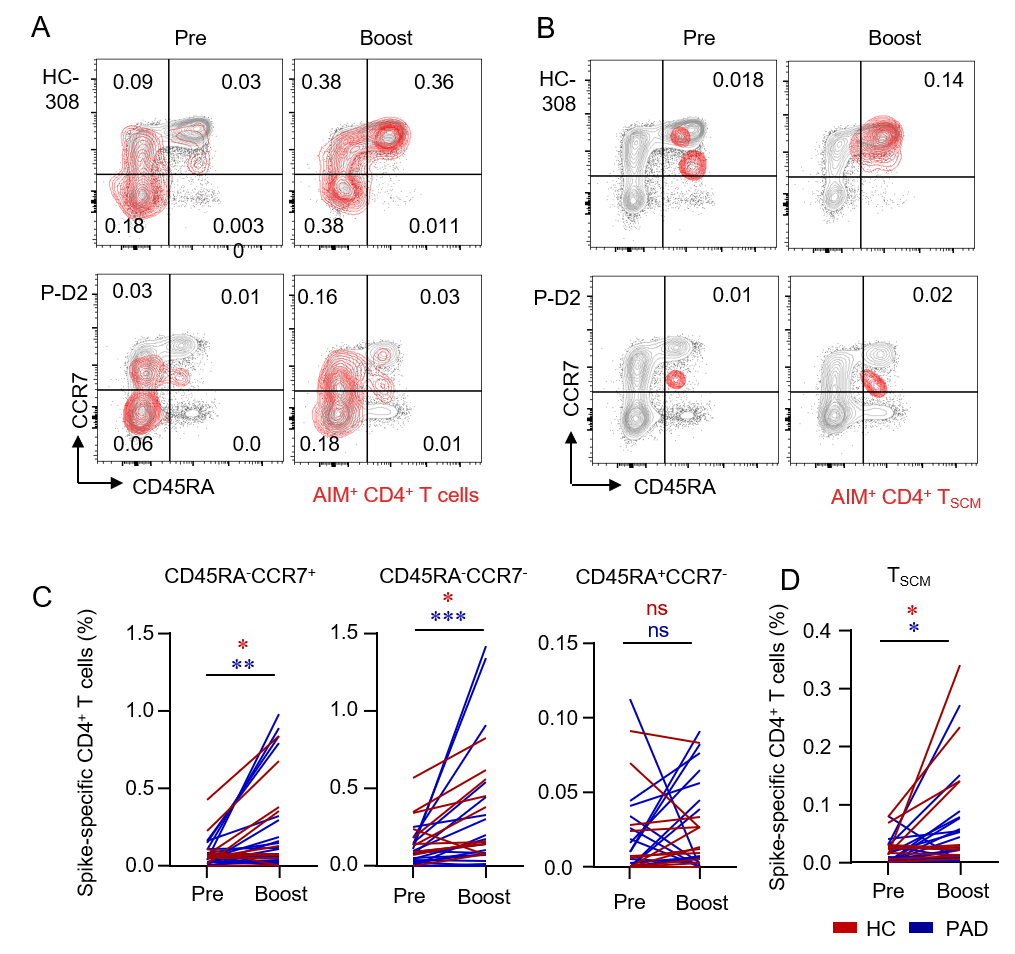


Supplementary Figure 12**. A third dose of inactivated vaccine expands spike-specific CD4^+^ T cell memory in patients with autoimmune disease and healthy controls**. (A) Representative flow cytometric plots for the AIM^+^ CD4^+^ T cells to identify the T cell memory subsets before and after the third dose of inactivated vaccine. (B) Representative flow cytometric plots for CD4^+^ T_SCM_ (CD4^+^CD45RA^+^CCR7^+^CD95^+^) cells before and after the third dose of inactivated vaccine. Red: CD4^+^ T_SCM_ cells, gray: total CD4^+^ T cells from the same donor. (C, D) Frequencies of spike-specific CD4^+^ memory T cell subsets (CD45RA^-^CCR7^+^, CD45RA^-^CCR7^-^, CD45RA^+^CCR7^-^, and T_SCM_) before and after the third dose of vaccine in HC and PAD. HC=11, PAD=18. *p<0.05, **p<0.01, ***p<0.001 by paired Wilcoxon test. ns: not significant.


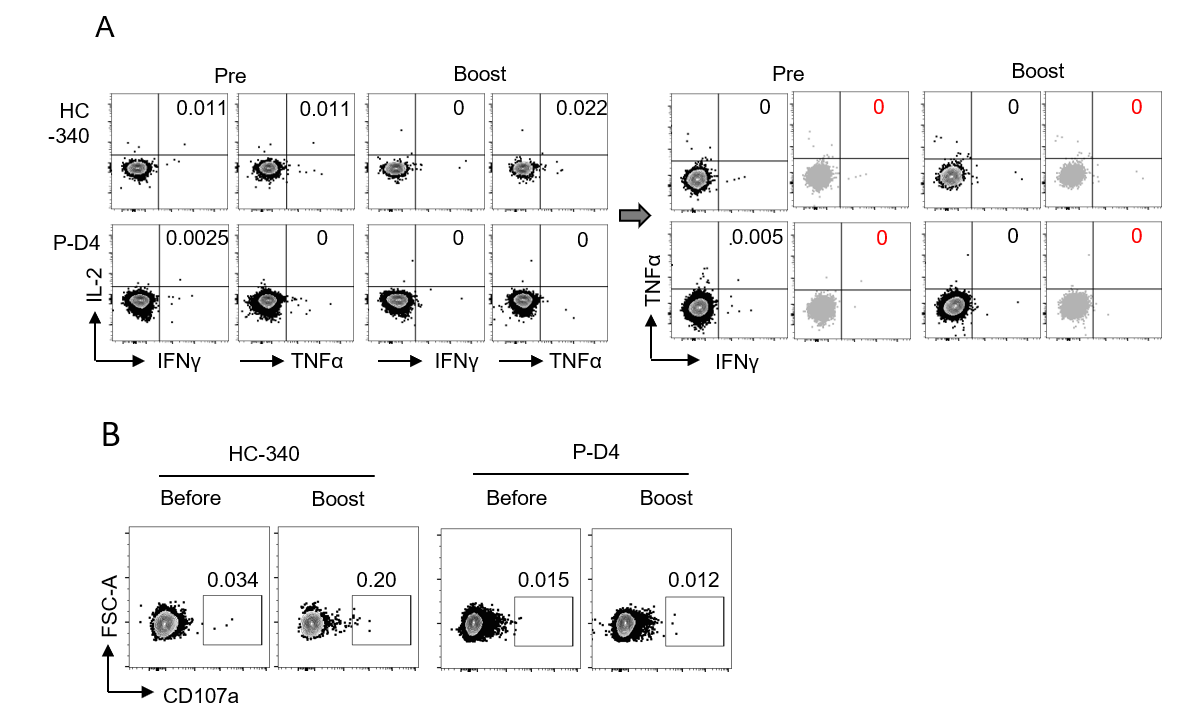


Supplementary Figure 13**. Quality control plots for a** **third dose of inactivated vaccine boosts spike-specific CD4^+^ T cell responses.** (A) DMSO representative plots of Figure 5I, as a quality control for boost cytokine^+^ CD4^+^ T cell response in HC and PAD. (B) DMSO representative plots of Figure 5L and Supplementary Figure 18D, as a quality control for boost cytotoxic CD4^+^ T cell response in HC and PAD.


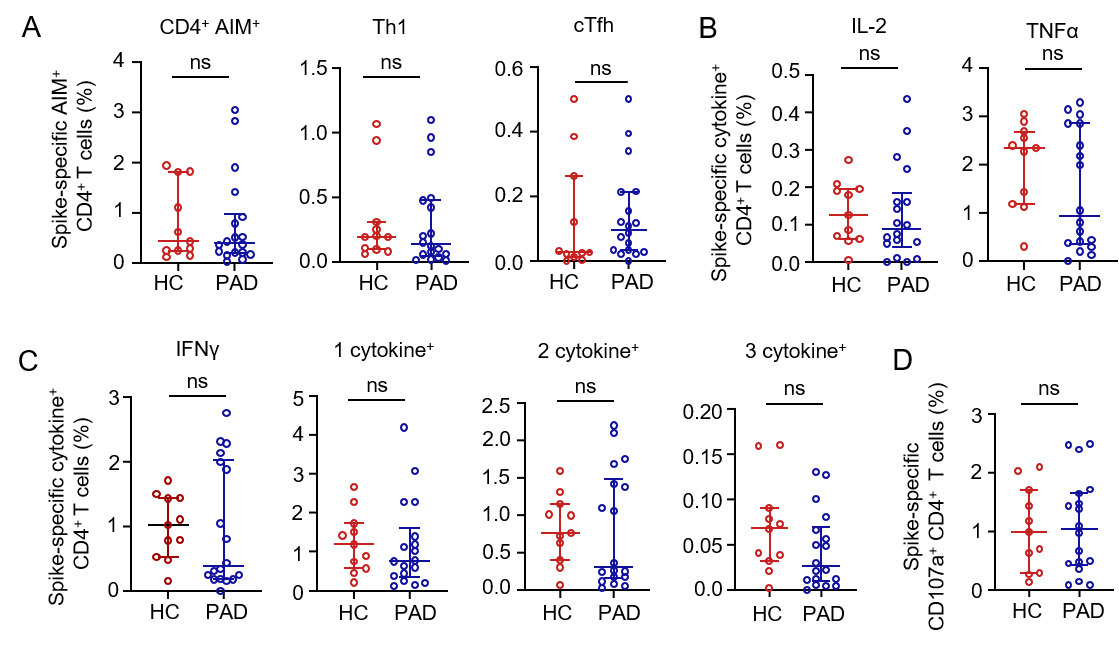
 Supplementary Figure 14. **Spike-specific CD4^+^ T cell responses after the third dose of vaccine in patients with autoimmune diseases and HC.** AIM expression and cytokine production in CD4^+^ T cells in response to spike peptide pools was measured and calculated as described in Supplementary Figure 2. (A) Frequencies of AIM^+^ CD4^+^ T cells subsets after the third dose in PAD and HC. (B) Frequencies of TNFα^+^ and IL-2^+^ CD4^+^ T cells after the third dose in PAD and HC. (C) Frequency of IFNγ^+^ ,1 cytokine^+^, 2 cytokine^+^, 3 cytokine^+^ CD4^+^ T cells after the third dose in PAD and HC. (D) Frequencies of CD107a^+^ CD4^+^ T cells after the third dose in PAD and HC. HC=11, PAD=18. Data are expression as median with interquartile range. ns: not significant by unpaired Wilcoxon test.


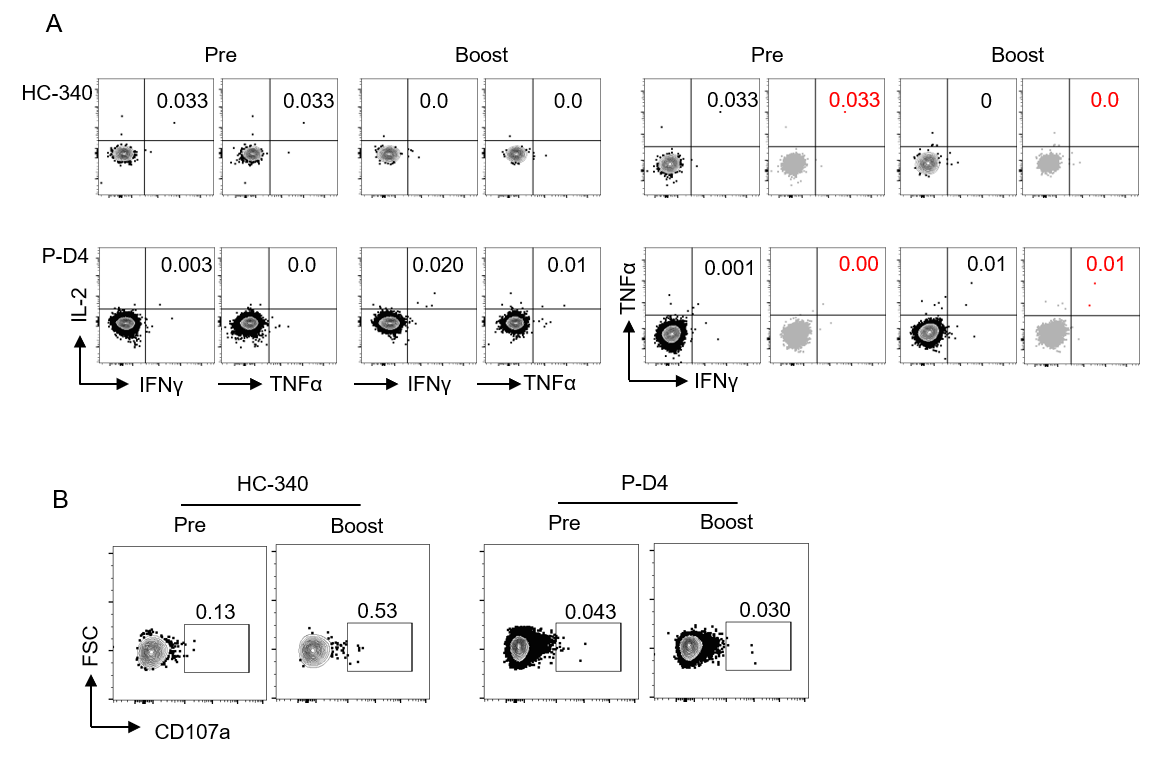


Supplementary Figure 15**. Quality control plots for a** **third dose of inactivated vaccine boosts spike-specific CD8^+^ T cell responses.** (A) DMSO representative plots of Figure 6D, as a quality control for boost cytokine^+^ CD8^+^ T cell response in HC and PAD. (B) DMSO representative plots of Figure 6H and Figure 19D, as a quality control for boost cytotoxic CD8^+^ T cell response in HC and PAD.


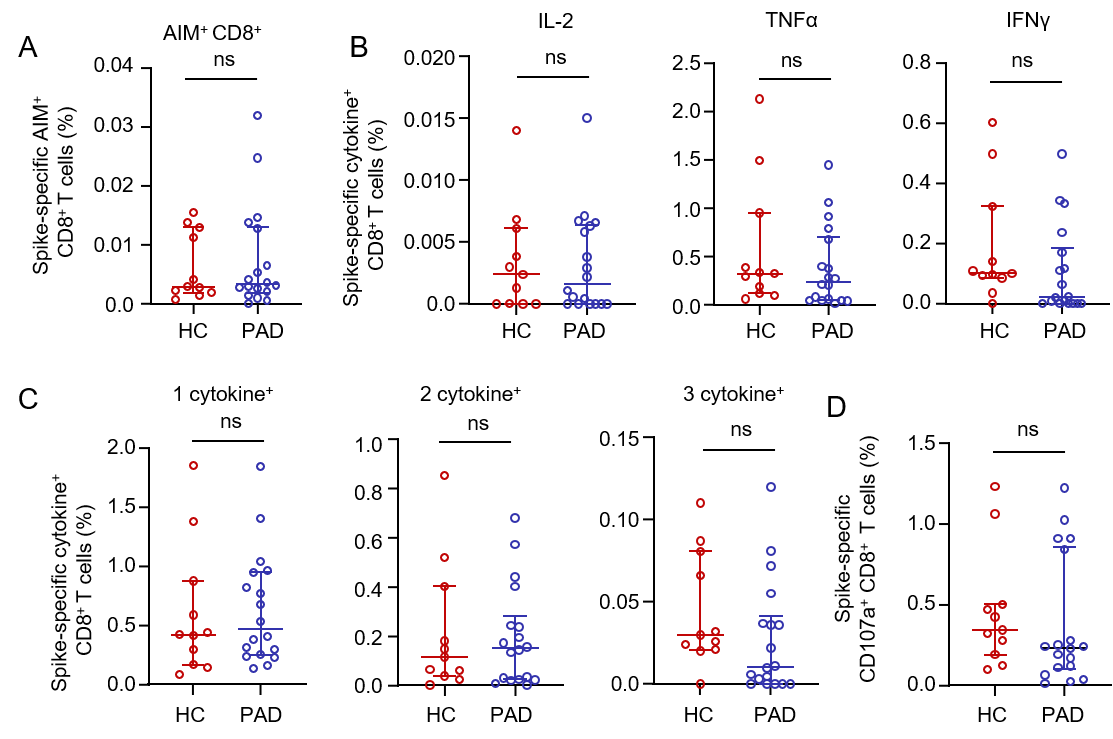


Supplementary Figure 16. **Spike-specific CD8^+^ T cell responses after the third dose of vaccine in patients with autoimmune diseases and HC**. AIM expression and cytokine production in CD8^+^ T cells in response to spike peptide pools was measured and calculated as described in Supplementary Figure 2. (A) Frequency of AIM^+^ CD8^+^ T cells subsets after the third dose in PAD and HC. (B) Frequencies of IFNγ^+^, TNFα^+^ or IL-2^+^ CD8^+^ T cells after the third dose in PAD and HC. (C) Frequencies of 1 cytokine^+^, 2 cytokine^+^, 3 cytokine^+^ CD8^+^ T cells after the third dose in PAD and HC. (D) Frequency of CD107a^+^ CD8^+^ T cells after the third dose in PAD and HC. HC=11, PAD=18. Data are expression as median with interquartile range. ns: not significant by unpaired Wilcoxon test.


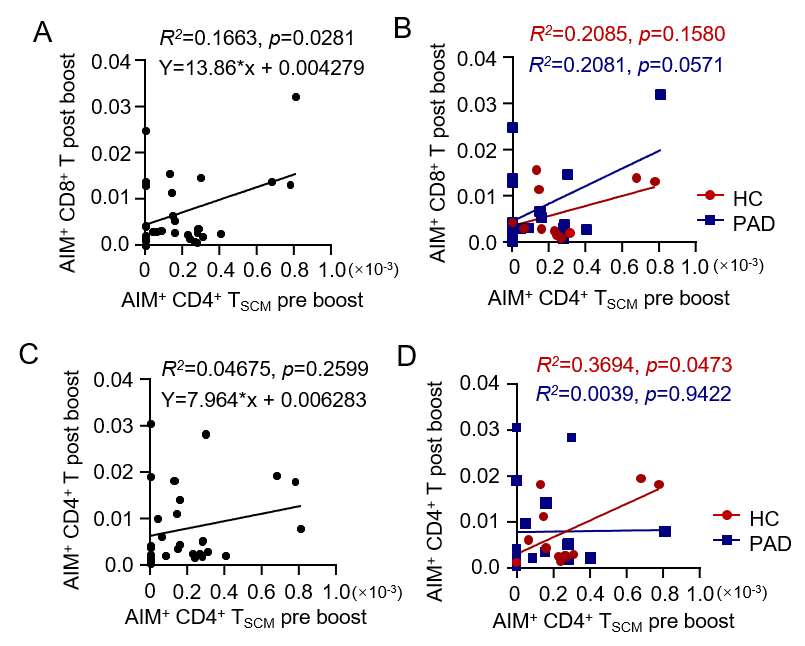


Supplementary Figure 17. **Correlation between the frequencies of CD4^+^ AIM^+^ T_SCM_** **cells before boost shot and frequency AIM^+^ T cells after boost shot.** (A, B) Correlation between the frequencies of CD8^+^ AIM^+^ T_SCM_ cells before boost shot and frequency AIM^+^ CD8^+^ T cells after boost shot. (C, D) Correlation between the frequencies of CD4^+^ AIM^+^ T_SCM_ cells before boost shot and frequency AIM^+^ CD4^+^ T cells after boost shot. Linear regression analysis was performed.


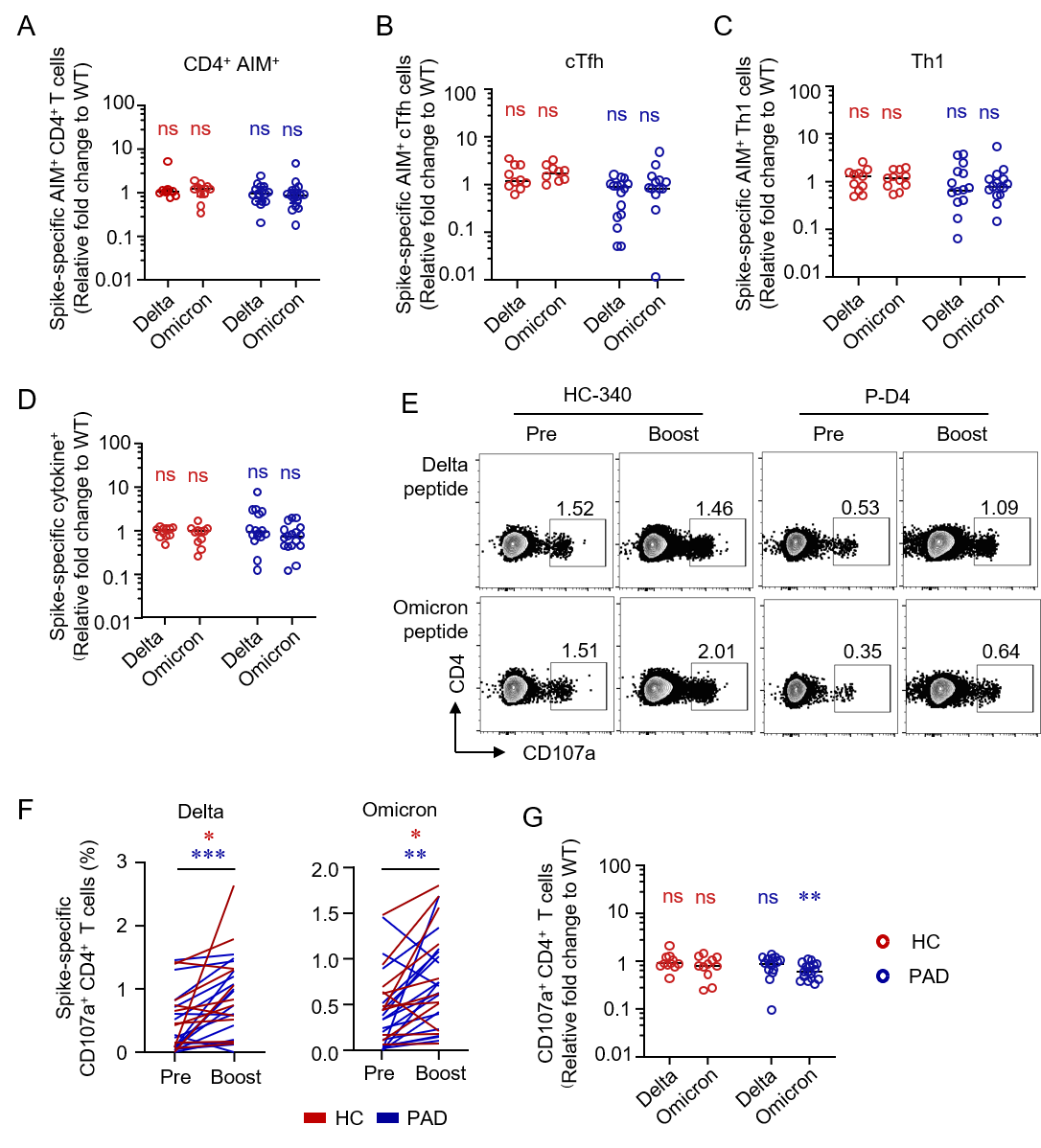


Supplementary Figure 18**. Spike-specific CD4^+^ T cell responses to Delta and Omicron by a third dose of vaccine.** (A) Relative fold-change of Delta and Omicron-responsive AIM^+^ CD4^+^ T cells to WT. (B, C) Relative fold-change of Delta and Omicron-responsive AIM^+^ cTfh and Th1 cells to WT. (D) Relative fold-change of cytokine^+^ CD4^+^ T cells to WT. (E, F) Representative flow cytometric plots and frequencies of the CD107a^+^ CD4^+^ T cells before and after the third dose. (G) Relative fold-change of CD107a^+^ CD4^+^ T to WT. Fold-change decreases for each variant was assessed by Wilcoxon signed rank T test compared with a hypothetical median of 1 in panels A-D, G. Data are expression as median with interquartile range. *p<0.05, **p<0.01 by paired Wilcoxon test in panel F. ns, not significant.


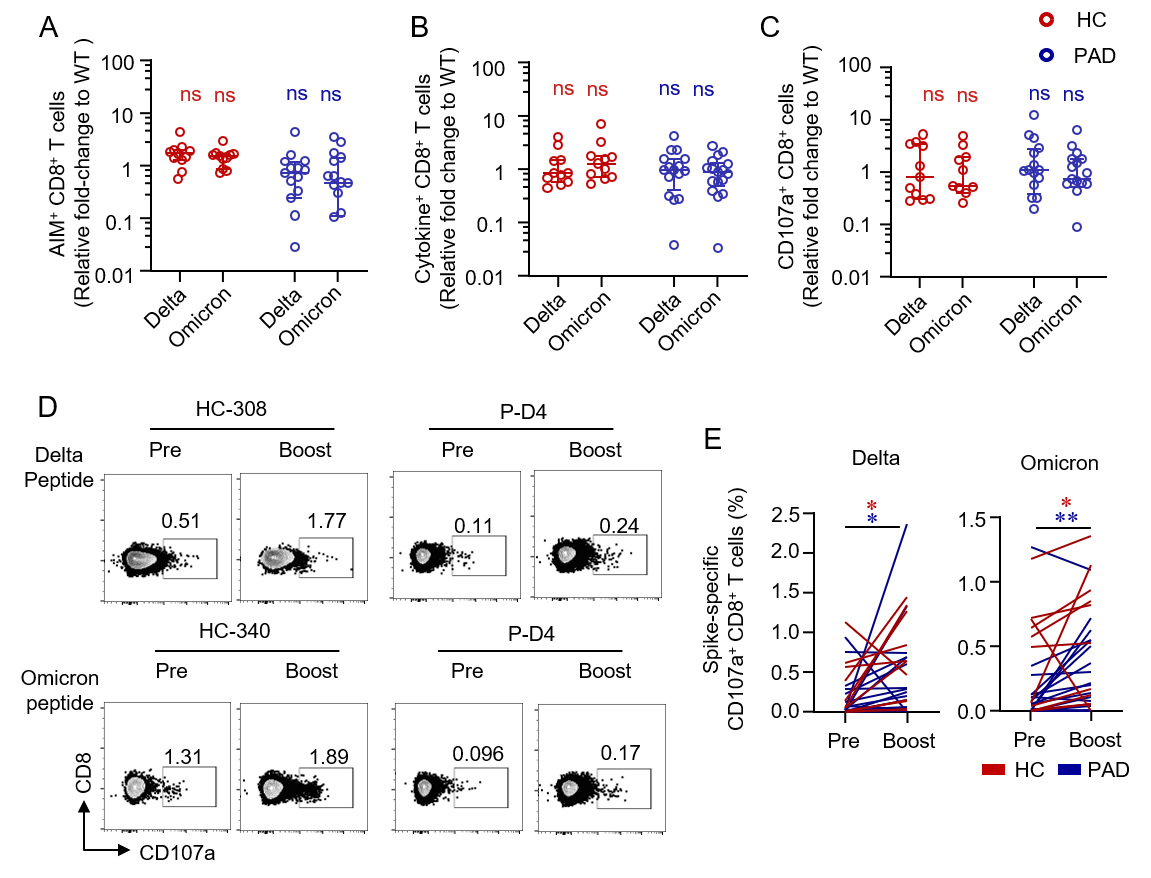


Supplementary Figure 19**. Spike-specific CD8^+^ T cell responses to Delta and Omicron by a third dose of vaccine.** (A) Relative fold-change of Delta and Omicron-responsive AIM^+^ CD8^+^ T cells to WT. (B) Relative fold-change of cytokine^+^ CD8^+^ T cells to WT. (C) Relative fold-change of CD107a^+^ CD8^+^ T to WT. (D, E) Representative flow cytometric plots and frequencies of the CD107a^+^ CD8^+^ T cells before and after the third dose of vaccine. HC=11, PAD=16 in Delta experiment, 17 in Omicron experiment. Fold-change decreases for each variant was assessed by Wilcoxon signed rank T test compared with a hypothetical median of 1 in panels A-C. Data are expression as median with interquartile range. *p<0.05, **p<0.01 by paired Wilcoxon test in panel E. ns, not significant.
